# The lipid peroxidation product 4-hydroxynonenal contributes to oxidative stress-mediated deterioration of the ageing oocyte

**DOI:** 10.1038/s41598-017-06372-z

**Published:** 2017-07-24

**Authors:** Bettina P. Mihalas, Geoffry N. De Iuliis, Kate A. Redgrove, Eileen A. McLaughlin, Brett Nixon

**Affiliations:** 1 Priority Research Centre for Reproductive Science, School of Environmental and Life Sciences, University of Newcastle, Callaghan, New South Wales Australia; 2 School of Biological Sciences, University of Auckland, Auckland, New Zealand

## Abstract

An increase in intraovarian reactive oxygen species (ROS) has long been implicated in the decline in oocyte quality associated with maternal ageing. Oxidative stress (OS)-induced lipid peroxidation and the consequent generation of highly electrophilic aldehydes, such as 4-hydroxynonenal (4-HNE), represents a potential mechanism by which ROS can inflict damage in the ageing oocyte. In this study, we have established that aged oocytes are vulnerable to damage by 4-HNE resulting from increased cytosolic ROS production within the oocyte itself. Further, we demonstrated that the age-related induction of OS can be recapitulated by exposure of germinal vesicle (GV) oocytes to exogenous H_2_O_2_. Such treatments stimulated an increase in 4-HNE generation, which remained elevated during *in vitro* oocyte maturation to metaphase II. Additionally, exposure of GV oocytes to either H_2_O_2_ or 4-HNE resulted in decreased meiotic completion, increased spindle abnormalities, chromosome misalignments and aneuploidy. In seeking to account for these data, we revealed that proteins essential for oocyte health and meiotic development, namely α-, β-, and γ-tubulin are vulnerable to adduction *via* 4-HNE. Importantly, 4-HNE-tubulin adduction, as well as increased aneuploidy rates, were resolved by co-treatment with the antioxidant penicillamine, demonstrating a possible therapeutic mechanism to improve oocyte quality in older females.

## Introduction

In humans, a finite number of female germ cells enter meiosis during embryonic life, only to become meiotically arrested in an extended prophase I, until recruitment into the growing follicle pool for ovulation decades later^1^. This dynamic process of oocyte growth and maturation must be tightly synchronized to ensure fidelity of the female germline. However, maternal ageing is accompanied by a precipitous decline in oocyte quality. This process is exemplified by dramatic, aged-associated increases in aneuploidy rates, and a concomitant elevation in the risk of miscarriage and birth defects^2–5^. Indeed, aneuploidy rates have been estimated to rise from an occurrence of approximately 2% of oocytes ovulated from women in their 20 s to between 35% and 50% of ovulated oocytes from women in their 40 s and 50 s, respectively^4–6^. While the mechanistic basis of age-associated elevation of aneuploidy rates is undoubtedly complex, several contributing factors have been identified. Chief among these appears to the loss of the cohesin protein complex that is responsible for tethering chromosomes together^7–10^. Additionally, chromosomal segregation is also perturbed by the inability of kinetochore microtubule attachments to be faithfully established^11^ in conjunction with reduced stringency of the downstream spindle assembly checkpoint^12–14^.

A ‘free radical theory’ has long been postulated as a leading causative agent underpinning the deterioration of oocyte quality with increasing maternal age^15, 16^. This hypothesis centres on the proposal that oocytes experience an accumulation of oxidative damage during the decades they spend in extended meiotic arrest while remaining metabolically active^15–18^. Indeed, despite their quiescent status, an oocyte’s mitochondria remain active in order to meet their basal metabolic demands^19^ and thus represent a potential source of intracellular ROS generation^20, 21^. This situation is exacerbated during maternal ageing owing to an attendant increase in oocyte mitochondrial dysfunction^22–28^. Among the numerous consequences of mitochondrial dysfunction is an increase in ROS generation as a by-product of electron leakage from the electron transport chain (ETC). Unfortunately, elevated concentrations of ROS also have the potential to damage mtDNA and proteins, leading to a state of auto-oxidation^29^. Concomitantly, the repair and defensive capacity of the oocyte has been reported to decrease with increasing maternal age. For instance, global transcriptomic analyses have revealed reduced expression of several antioxidant enzymes in ovulated oocytes recovered from aged mice and human donors^4, 30, 31^. Collectively, such changes place the aged oocyte at increased risk of oxidative stress.

The role of oxidative stress (OS) in perpetuating the ageing phenotype is further supported by an increase in ROS detected in the follicular fluid of women of advanced maternal age^15, 16^. In particular, hydrogen peroxide (H_2_O_2_) has been implicated as a marker for human ovarian ageing^32^ and an increase in free-radical activity has been correlated with a decrease in, *in vitro* fertilisation (IVF) success rates^33^. Furthermore, elevated levels of ROS have been associated with a decrease in meiotic completion^34, 35^ and age-associated phenotypes including altered spindle microtubules, chromosome misalignment^36–38^, aneuploidy^15, 39^ and diminishing embryo developmental potential^32, 40–44^. These combined effects ultimately culminate in reduced pregnancy rates. Despite compelling clinical evidence that age-dependent accumulation of OS is, at least in part, responsible for the associated decline in oocyte quality, much of the mechanistic basis of its action remains to be determined.

It has been well established that the induction of cellular OS initiates the peroxidation and destruction of lipids. Glycolipids, phospholipids and cholesterol are all vulnerable to this damaging and potentially lethal process of electron scavenging^45–47^, with ω-6 polyunsaturated fatty acids such as arachidonic and linoleic acids serving as primary targets^48, 49^. During the peroxidation cascade, oxygen insertion and the ensuing hydrogen abstraction, result in the formation of lipid peroxyl radicals and hydroperoxides^45^. Downstream of these events, a suite of electrophilic aldehydes, including 4-hydroxynonenal (4-HNE), are formed as secondary products of the peroxidation cycle. These reactive aldehyde species are able to modify proteins by preferential covalent adduction of the amino acids cysteine, histidine and lysine^50, 51^. These lipid aldehyde adducts can subsequently interfere with protein function in a number of ways including the induction of protein crosslinking, structural perturbation and protein aggregation^52–56^. In the majority of cell types, physiological levels of OS and the ensuing damage elicited by lipid peroxidation, can be countered through the activation of antioxidant defences and/or stringent repair pathways. However, the exposure of cells to concentrations of OS that overwhelm their protective capacity, can lead to a suite of pathological changes that culminate in the eventual loss of cell viability^57^.

As one of the most abundant and cytotoxic of the lipid peroxidation products, 4-HNE has recently been demonstrated to cause pronounced dysfunction in the male germline^58–61^. Indeed, 4-HNE is readily able to adduct and alter the function of several vulnerable targets within the human sperm proteome. Among the most prevalent of these proteins are α-tubulin^58^, the molecular chaperone HSPA2^59^ and succinate dehydrogenase (SDHA). The resulting dysregulation of these proteins is known to compromise sperm-oocyte recognition and promote electron leakage from the electron transport chain, thus exacerbating the level of OS experienced by the spermatozoa^60^. Importantly, SDHA has also proven to be a conserved target for 4-HNE adduction in postovulatory aged metaphase II (MII) mouse oocytes, thus implicating the aldehyde in deterioration of oocyte quality^29^. Such findings take on added significance in view of the increase in 4-HNE generation observed within the ovarian interstitial tissue of ageing mice^62^. Taken together, such evidence supports the tenet that the ageing oocyte is exposed to elevated OS and the associated lipid peroxidation by-products. Surprisingly however, the effects of such insults on oocyte meiosis have yet to be directly investigated. Indeed, despite the recognition that key meiotic proteins such as α-tubulin, are susceptible to oxidative lesions in the male germline^58^, to the best of our knowledge there are currently no equivalent reports linking aldehyde adduct formation to the dysregulation of meiosis in the female gamete.

In this study, we explore the hypothesis that the precipitous loss of oocyte quality experienced during maternal ageing is, at least in part, attributed to the accumulation of oxidative damage. More specifically, we predict that one of the key causative agents in this aetiology is the highly reactive lipid aldehyde, 4-HNE, which is generated as a by-product of lipid peroxidation and has the potential to covalently adduct to vulnerable oocyte proteins that control the fidelity of meiotic divisions. By improving our understanding of the mechanisms by which oocyte quality declines with age such studies should help inform the development of therapeutic interventions for women choosing to delay child bearing until their later reproductive years.

## Results

### Maternally aged oocytes accumulate more cytosolic ROS and experience elevated 4-HNE exposures

The lipid-aldehyde, 4-HNE, represents one of the primary by-products of lipid peroxidation cascades and is a major contributor to pathologies generated under conditions of OS^63, 64^. To investigate the possibility of 4-HNE as a causative intermediate of the age-related decline in oocyte quality, our initial experiments sought to establish whether this aldehyde is generated within ovarian oocytes and their follicular environment. For the purpose of these studies we employed a C57Bl/6xCBA F1 hybrid cross (F1) mouse model since the age-dependent oocyte deterioration previously documented in this strain, mirrors that which occurs in humans. Indeed, by 14 months of age (equivalent to approximately 40 years in humans) female F1 mice display a significant increase in chromosome segregation errors occurring in approximately half of their oocytes^2–5, 65^.

Immunohistochemical analysis confirmed the presence of substantial 4-HNE within the ovarian tissue of both young (4–6 weeks) and aged (14 months) F1 mice. Although 4-HNE was detected in the majority of ovarian cell types, intense labelling was detected within the cytosol of oocytes in secondary and antral follicles (Fig. S1).

**Figure 1:**
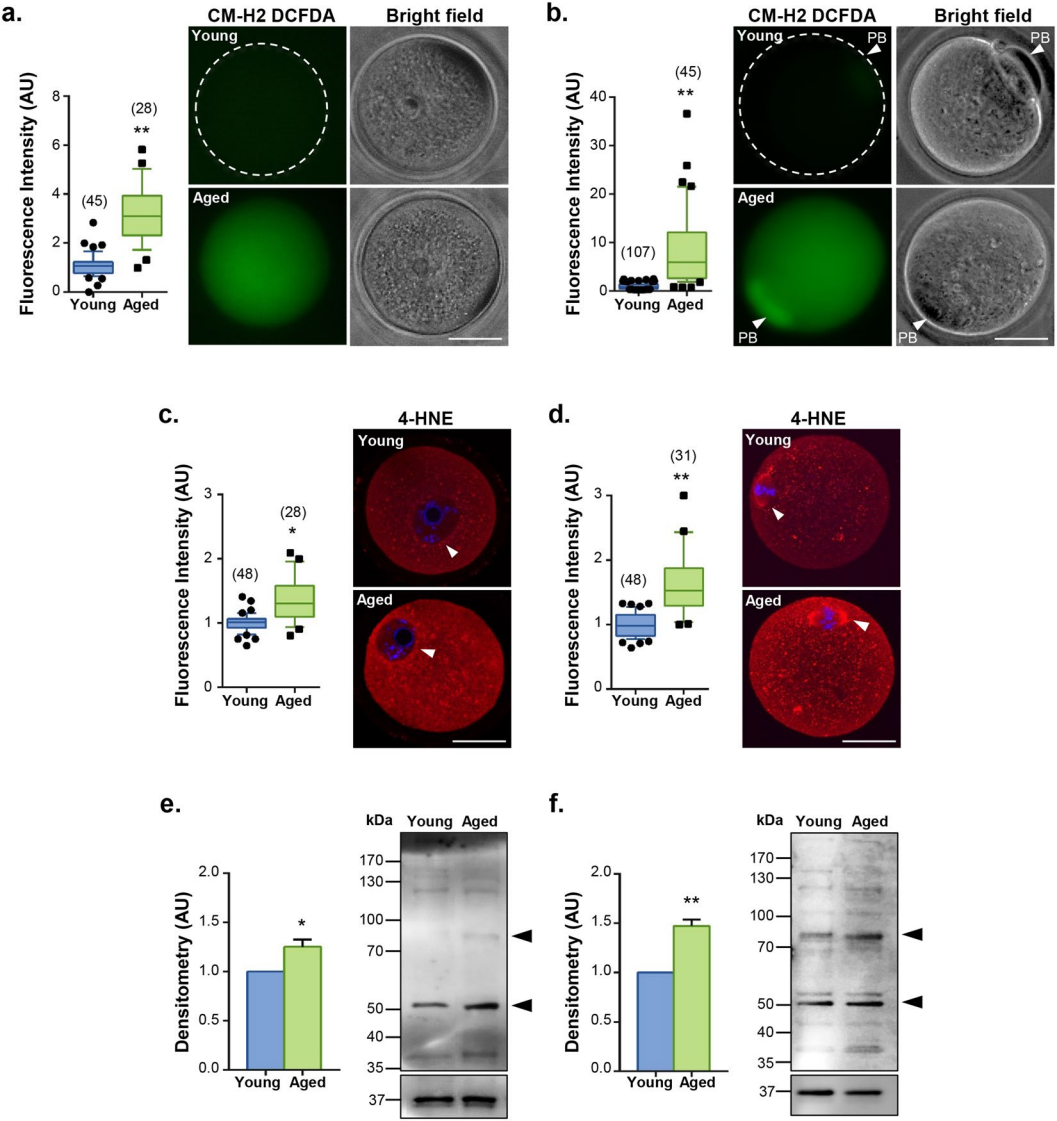
Oocytes from aged mice carry a higher oxidative burden and have an increase in 4-HNE accumulation. GV oocytes were collected from un-primed young animals (4 to 6 weeks) and aged animals (14 months) or underwent IVM to MII. (**a**) GV and (**b**) MII oocytes from aged animals displayed elevated levels of cytosolic ROS as indicated by labelling with the CM-H2 DCFDA probe (green). White arrows indicate the polar body (PB). Scale bar = 20 μm. (**c**) An increase in the accumulation of 4-HNE was detected using immunocytochemistry against 4-HNE (red) in both GV and (**d**) MII oocytes. White arrows indicate the nuclear envelope and spindle, respectively. Nuclei were counterstained with Hoechst (blue). Scale bar = 20 μm. Box and whisker plots show means as the centreline, box as the 25–75th percentiles and whiskers as the 10–90th percentiles. Ageing assays were performed with n = 4 mice, with each mouse contributing a minimum of 7 oocytes and representing an independent technical replicate. (**e**) An increase in the 4-HNE adduction in aged oocytes was also confirmed *via* 4-HNE immunoblotting, which revealed an increase in the band intensity of the majority of 4-HNE modified proteins in aged GV and (**f**) MII oocyte lysates. Black arrows indicate the predominate proteins at approximately 80 and 55 kDa. For all samples, the pixel intensity of the entire lane was analysed by densitometry. Immunoblots were stripped and re-probed with GAPDH as a loading control. Error bars represent SEM. Statistical analyses were performed using Student’s t-test, *p ≤ 0.05 and **p ≤ 0.01. Immunoblots were performed in biological and technical triplicate using 100 oocytes per lane pooled from a minimum of 3 young animals and between 12 to 15 aged animals.

In view of these data, we next focused our attention on 4-HNE expression within isolated oocytes to examine whether pathological phenotypes associated with the ageing oocyte could, at least in part, be attributed to ROS induced 4-HNE production. As cumulus cells possess their own oxidative defence capacity^66, 67^, we elected to denude oocytes prior to treatment. This strategy reflected our main objective of eliciting an oxidative response to elucidate the role of 4-HNE in the deterioration of oocyte quality. Interestingly, a statistically significant, 3.2- and 8.2-fold increase in CM-HDCFDA fluorescent labelling of intra-cellular ROS was observed in both germinal vesicle (GV) (*p* ≤ 0.0045; Fig. 1a) and in MII oocytes including polar body (PB) (*p* ≤ 0.0025; Fig. 1b) from young versus those of aged mice. These increased levels of cytosolic ROS were accompanied by a significant, 1.4- and 1.7-fold elevation in the fluorescent labelling of 4-HNE in young versus aged GV (*p* ≤ 0.0407; Fig. 1c) and MII oocytes (*p* ≤ 0.0021; Fig. 1d). In this context, punctate 4-HNE labelling was readily detected throughout the cytosol of oocytes (both GV and MII) but was observed to notably increase in the vicinity of the nuclear envelope of GV oocytes and in association with the meiotic spindle of MII oocytes. Immunoblotting analyses strengthened these observations, revealing a 1.3- to 1.5-fold increase in the labelling intensity of 4-HNE adducted proteins ranging in size from approximately 37 to 200 kDa in GV and MII aged oocytes, respectively (*p* ≤ 0.0252, 0.0018; Fig. 1f,g). This increased labelling was particularly evident in the most predominant bands of approximately 55 and 80 kDa. These data further implicate 4-HNE generation and the ensuing protein adduction as a contributor to the decline in oocyte quality that accompanies advanced maternal age.

### H_2_O_2_ induced lipid peroxidation culminates in an increase in 4-HNE production

**Figure 2:**
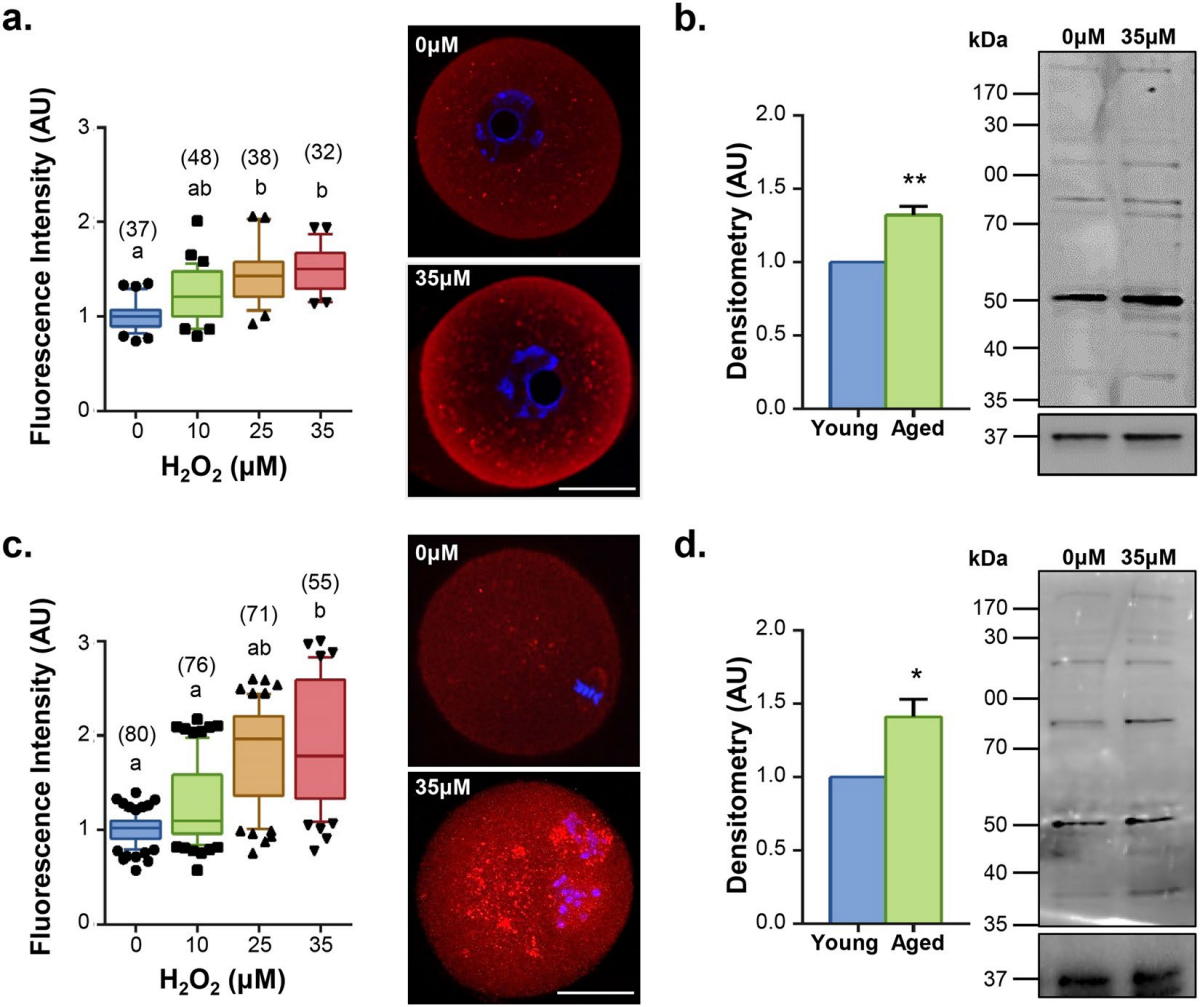
OS induces the generation of 4-HNE. Immunocytochemistry and immunoblotting were used to determine whether an increase in 4-HNE modifications could be elicited by H_2_O_2_ treatment. For immunocytochemical analysis, GV oocytes were treated with 10, 25 or 35 μM H_2_O_2_ for 1 h and immediately fixed or underwent IVM for subsequent fixation at MII. Both (**a**) GV (*p* ≤ 0.0440) and (**c**) MII oocytes (*p* ≤ 0.0280) showed a dose-dependent increase in 4-HNE expression (red). Nuclei were counterstained with Hoechst (blue). Scale bar = 20 μm. Box and whisker plots shows mean as the centreline, box as the 25–75th percentiles and whiskers as the 10–90th percentiles. Immunocytochemistry was performed with three biological replicates with each replicate containing between 10–30 oocytes pooled from a minimum of three animals. (**b**) An increase in the adduction profile of 4-HNE following H_2_O_2_ treatment was also confirmed in both GV and (**d**) MII oocytes *via* 4-HNE immunoblotting, again indicating an increase in 4-HNE modification at 35 μM H_2_O_2_. Pixel intensity was calculated across entire lanes for the purpose of densitometry analysis. Immunoblots were stripped and re-probed with GAPDH as a loading control. Error bars represent SEM. Statistical analyses were performed using Student’s t-test, *p ≤ 0.05 and **p ≤ 0.01. Immunoblots were performed in biological and technical triplicate using 100 oocytes per lane pooled from a minimum of three animals.

Given the readily detectable increase in ROS and 4-HNE observed in aged oocytes, we next sought to establish a link between OS and the induction of lipid peroxidation and subsequent 4-HNE generation in oocytes. H_2_O_2_ was selected as the initial oxidant as it has been confirmed to significantly elevate 4-HNE production in gametes^59^ and identified as a marker for oocyte maternal ageing^32^. For this purpose, GV and MII oocytes were exposed to increasing concentrations of H_2_O_2_ prior to assessing 4-HNE generation and protein adduction within these cells. Immunocytochemical analysis revealed a significant, approximate 1.4- to 1.5-fold increase in 4-HNE labelling of GV oocytes after a 1 h exposure to as little 35 µM H_2_O_2_, respectively compared to that of untreated controls (*p* ≤ 0.0440; Fig. 2a). Importantly, these concentrations of H_2_O_2_ fall within the physiological range experienced by cells under conditions of OS, with concentrations of up to 50 µM H_2_O_2_ having been reported in biological fluids^68^.

Elevated 4-HNE expression was documented throughout the cytosol of H_2_O_2_-treated oocytes and was again characterised by the labelling of numerous punctate foci. These data were validated *via* immunoblot analysis, which confirmed a 1.3-fold increase in 4-HNE labelling intensity of all putative 4-HNE modified proteins detected in the GV oocyte population (*p* ≤ 0.0061; Fig. 2b). Importantly, the dose-dependent increase in both 4-HNE expression and protein adduction elicited by H_2_O_2_ failed to be resolved during *in vitro* oocyte maturation (IVM). In this regard, the resulting population of MII oocytes retained a cytosolic profile of 4-HNE labelling that was at least 1.3-fold more intense than that of untreated oocytes at all concentrations of H_2_O_2_ examined (*p* ≤ 0.0280; Fig. 2c). Similarly, the 4-HNE labelling of MII oocytes was again distributed across a similar profile of proteins to those detected in immunoblots of GV oocyte lysates, representing a 1.4-fold increase in 4-HNE adduction upon H_2_O_2_ treatment (*p* ≤ 0.0248; Fig. 2d). Moreover, noticeable chromosome misalignment in treated oocytes prompted us to investigate meiosis further (Fig. 2c).

### 4-HNE generation negatively impacts on meiotic competency

Having established that the levels of 4-HNE are significantly elevated upon exposure of oocytes to H_2_O_2_, we next sought to determine the impact of this signature of enhanced OS on the meiotic competency of oocytes. In addition to H_2_O_2_ (10–100 µM), this study incorporated the use of an exogenous 4-HNE treatment (5–50 µM), with concentrations of both oxidants being selected to approximate those of moderate levels of physiological OS^68, 69^. In the context of 4-HNE, it has been suggested that this electrophile accumulates in membranes at concentrations of between 10 μM to 5 mM following oxidative insult^57^. As with the former studies, GV oocytes were subjected to the appropriate treatment prior to undergoing *in vitro* maturation.

**Figure 3:**
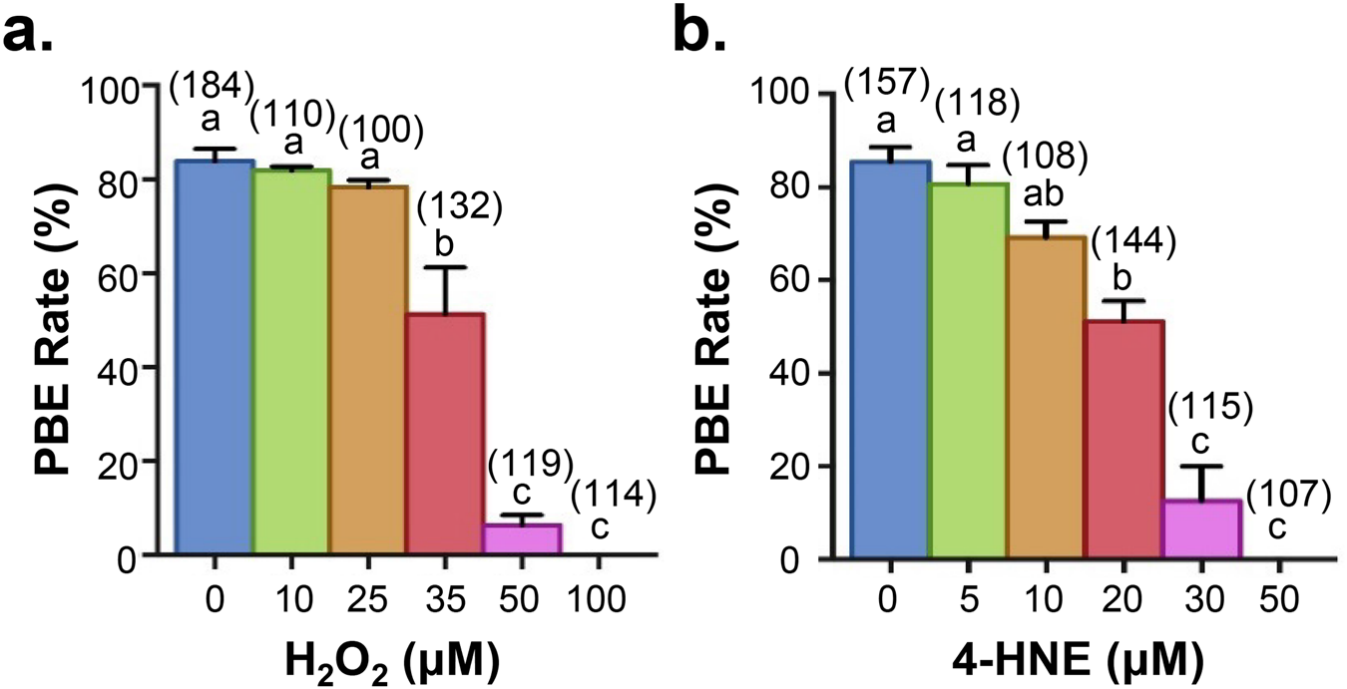
Acute exposure to H_2_O_2_ and 4-HNE at GV stage causes a dose-dependent decrease in meiotic completion during IVM. Oocytes at GV stage were treated with either H_2_O_2_ for 1 h or 4-HNE for 2 h prior to IVM for 16 h. MII oocytes were identified by the presence of a polar body. (**a**) A dose-dependent decrease in polar body extrusion (PBE) was observed after H_2_O_2_ (*p* ≤ 0.0011) and (**b**) 4-HNE (*p* ≤ 0.0013) treatments. Error bars represent SEM. IVM experiments were performed with five biological replicates with each replicate containing between 20–50 oocytes pooled from a minimum of three animals.

Both H_2_O_2_ and 4-HNE treatments were able to compromise oocyte meiosis. Indeed, both forms of oxidative insult elicited a potent, dose-dependent suppression of polar body extrusion (PBE) rates, decreasing from between 84–85% in untreated controls to 50–51% at moderate doses (35 µM; H_2_O_2_ 20 µM 4-HNE) before completely eliminating PBE at the highest doses used in this study (100 µM H_2_O_2_; 50 µM 4-HNE) (*p* ≤ 0.0011, 0.0013; Fig. 3a,b). On the basis of these collective data, subsequent studies were conducted using maximum concentrations of up to 35 µM H_2_O_2_ and 20 µM 4-HNE; doses at which meiosis, but not cell vitality, were significantly compromised (Fig. S3e,f).

### Oocyte quality markedly decreased after acute exposure to H_2_O_2_ and 4-HNE

**Figure 4:**
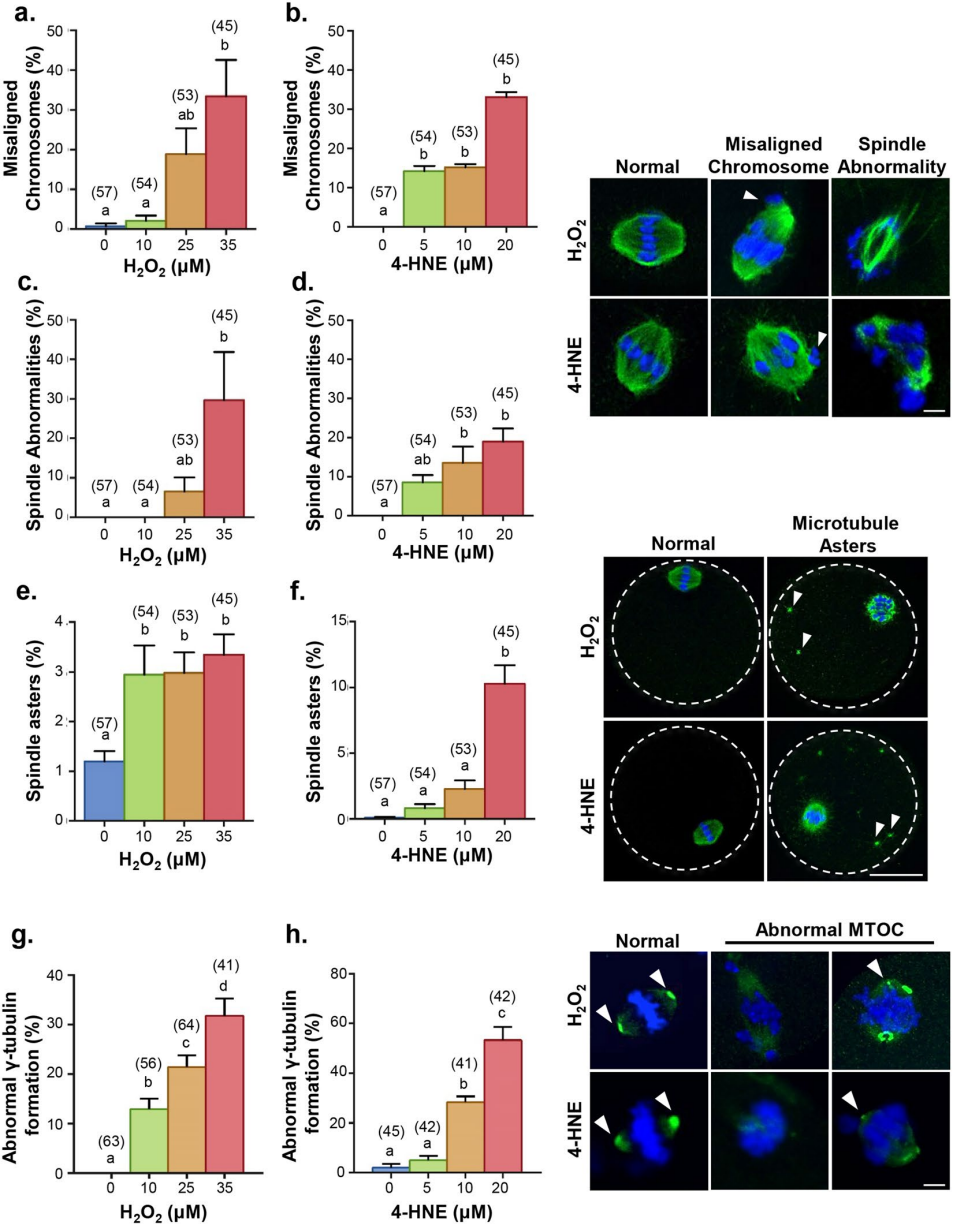
Acute exposure to H_2_O_2_ and 4-HNE at prophase I arrest led to a reduction in MII spindle integrity. GV oocytes were treated with either with 10, 25 and 35 μM H_2_O_2_ for 1 h or 5, 10 and 20 μM 4-HNE for 2 h. Following treatment, oocytes underwent IVM for fixation at MII to observe spindle and chromosome integrity using α-tubulin immunofluorescence (green) and the nuclear stain Hoechst (blue). (**a**) An increase in chromosome misalignment was observed at 35 µM H_2_O_2_ (*p* ≤ 0.0044) and (**b**) 5, 10 and 20 µM 4-HNE treatment (*p* ≤ 0.0001). White arrows point to misaligned chromosome(s). (**c**) Additionally, an increase in spindle anomalies was also documented at concentrations of 35 µM H_2_O_2_ (*p* ≤ 0.0131) and (**d**) 10 and 20 µM 4-HNE (*p* ≤ 0.0388). Scale bar = 5 μm. (**e**) An increase in the number of microtubule asters was also identified at 10, 25 and 35 μM H_2_O_2_ (*p* ≤ 0.0188) and (**f**) 20 μM 4-HNE (*p* ≤ 0.0001). Scale bar = 20 μm. (**g**) Lastly, a dose-dependent increase in abnormal formation of the MTOC was identified at 10, 25 and 35 μM H_2_O_2_ (*p* ≤ 0.038) and (**h**) 10 and 20 µM of 4-HNE (*p* ≤ 0.0089) using γ-tubulin immunofluorescence (green). Nuclei were counterstained with Hoechst (blue). Scale bar = 5 μm. Error bars represent SEM. Immunocytochemistry was performed with three biological replicates with each replicate containing between 10–30 oocytes pooled from a minimum of three animals.

To further investigate the consequences of acute H_2_O_2_ and 4-HNE exposure on oocyte quality we next assessed the integrity of the MII spindle, as a surrogate measure of embryonic potential^70^, and noted a dose-dependent decrease in the percent of normal spindles. Indeed, both H_2_O_2_ and 4-HNE treatments precipitated a significant increase in the percentage of oocytes bearing chromosomal misalignments; up from ≤1% in untreated controls to approximately 33% in oocytes exposed to either 35 µM H_2_O_2_ (Fig. 4a) or 20 µM 4-HNE (Fig. 4b). Immunofluorescent staining of α-tubulin revealed that these defects were accompanied by a significant increase in both meiotic spindle abnormalities (Fig. 4c,d) and the formation of microtubule spindle asters (Fig. 4e,f). Such pronounced alterations to microtubule dynamics extended to deformities in the microtubule organising centre (MTOC), representing the major site of microtubule nucleation. In this context, immunofluorescent analysis of γ-tubulin demonstrated a significant, dose-dependent increase in aberrant formation of the MTOC; such defects were absent in untreated controls yet feature prominently among 32% and 52% of oocytes exposed to 35 µM H_2_O_2_ and 20 µM 4-HNE, respectively (Fig. 4g,h). The dominant MTOC abnormalities included displacement of γ-tubulin foci such that immunofluorescent labelling of the protein appeared as weak, punctate staining extending throughout the spindle poles, and/or in the vicinity of malformed spindle caps.

**Figure 5:**
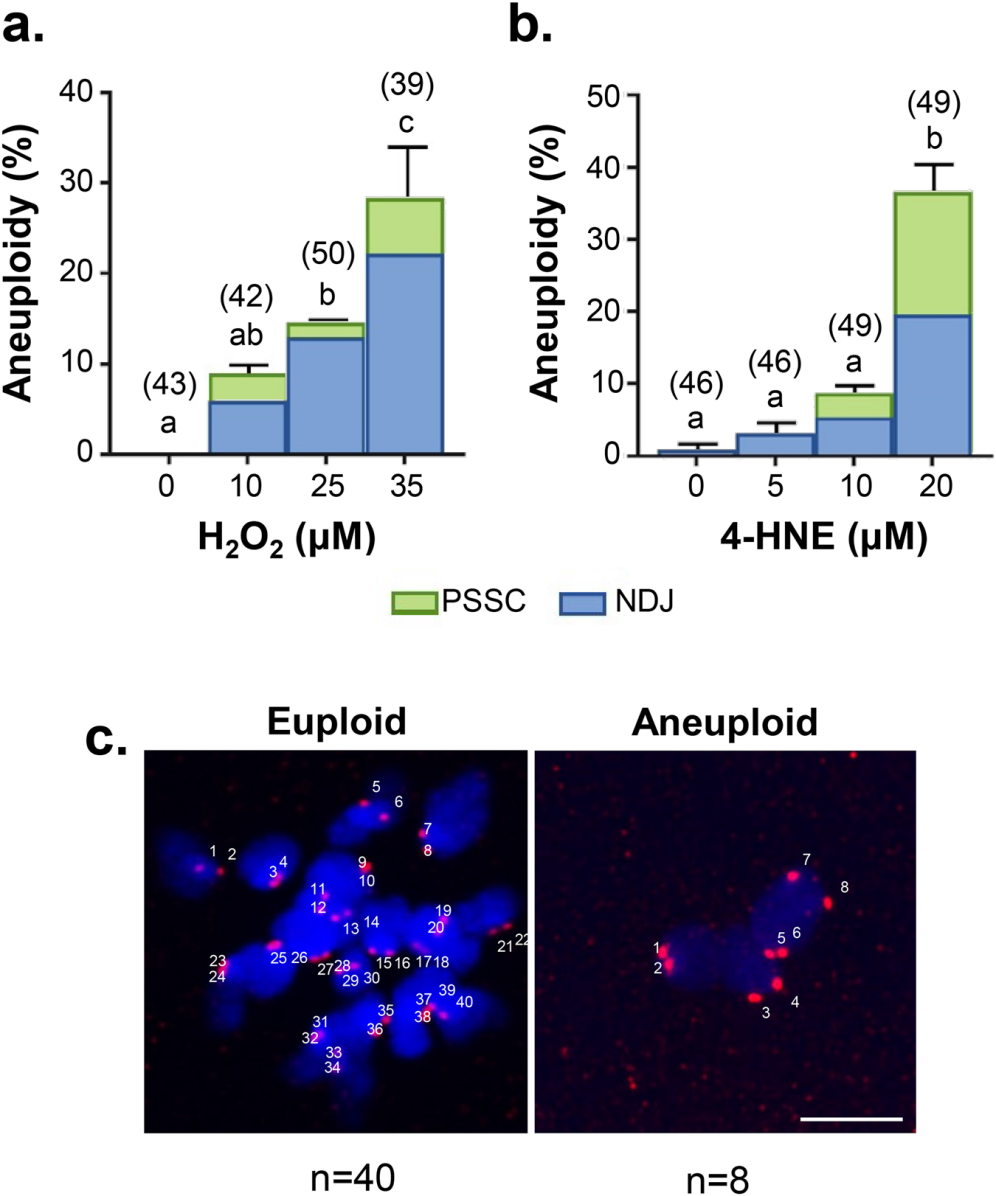
Acute exposure to H_2_O_2_ and 4-HNE at prophase I arrest led to a significant increase in aneuploidy in MII oocytes following IVM. GV oocytes were treated with either 10, 25 and 35 μM H_2_O_2_ for 1 h or 5, 10 and 20 μM 4-HNE for 2 h. Following treatment, oocytes underwent IVM, spindles were collapsed and oocytes were fixed. Kinetochores were immuno-stained (red) and the chromosomes were stained with Hoechst (blue). Kinetochores of each individual oocyte were counted. (**a**) An increase in aneuploid oocytes was detected at 25 and 35 μM H_2_O_2_ (*p* ≤ 0.0359) and (**b**) 20 μM 4-HNE (*p* ≤ 0.0001) in the form of both PSSC (green) and NDJ (blue). (**c**) Representative images are presented, indicating how aneuploidy was scored. Scale bar = 5 μm. Error bars represent SEM. Aneuploidy counts were performed with three biological replicates with each replicate comprising between 10–30 oocytes pooled from a minimum of three animals.

Given the fundamental role that the oocyte spindle holds in ensuring the faithful segregation of chromosomes^71^, it was reasoned that the loss of integrity that accompanies OS would elevate the risk of aneuploidy in these cells. Consistent with this notion, acute exposure of GV stage oocytes to either H_2_O_2_ or 4-HNE induced a significant, dose-dependent increase in the percentage of MII oocytes presenting with aneuploidy following IVM. Indeed, from modest basal levels of ≤1%, the number of aneuploid oocytes increased to account for 28% of those cells exposed to 35 µM H_2_O_2_ (Fig. 5a; *p* ≤ 0.0395) and 37% of oocytes treated with 20 µM 4HNE (*p* ≤ 0.0001; Fig. 5b) as represented by Fig. 5c. Oocyte aneuploidy can result from either the premature separation of sister chromatids (PSSC) whereby individual chromosomes are gained or lost, or bivalent nondisjunction (NDJ) whereby complete chromosome pairs are gained or lost^65^. Indeed, in our analysis we recorded both forms of aneuploidy in response to H_2_O_2_ and 4-HNE treatments.

### Tubulins are targeted for adduction by 4-HNE in oocytes and this adduction is more prevalent in aged oocytes

To begin to investigate the potential mechanism(s) by which OS reduces oocyte quality, we focused on the identification of the predominant 55 kDa protein(s) targeted for adduction by 4-HNE (Fig. 1f,g) using an LC-MS/MS interface. This strategy returned high confidence identification of multiple peptides mapping to either α- and/or β-tubulin (Table S1). Since α- and β-tubulin isoforms constitute key structural elements of the meiotic spindle, which is subject to considerable perturbation following oxidative insult, we performed detailed validation of the susceptibility of these proteins to 4-HNE adduction using a combination of co-localisation, immunoprecipitation and proximity ligation assays. Although it was not identified by MS analysis, we also elected to examine the gamma-tubulin isoform owing to the fact that it constitutes the principal structural component of the MTOC, which itself is significantly impacted by OS.

**Figure 6:**
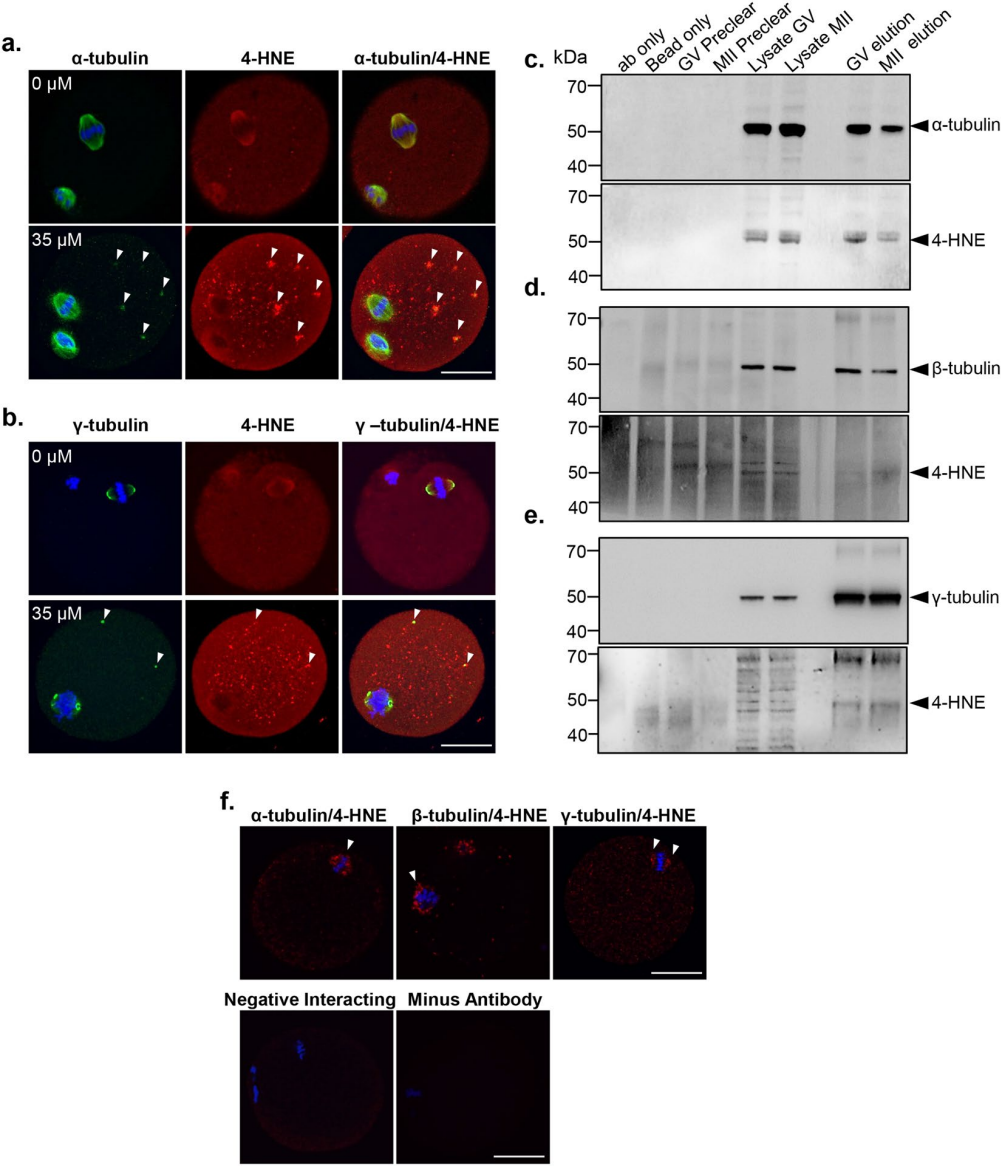
Examination of α-, β- and γ-tubulin/4-HNE interaction in GV and MII oocytes. Co-localisation, immunoprecipitation and PLA experiments were employed to examine the interaction between tubulins and 4-HNE. (**a**) For co-localisation, GV oocytes were untreated or treated with 35 μM H_2_O_2_ for 1 h and then underwent IVM in preparation for fixation at MII. α-tubulin (green) co-localised with 4-HNE (red) at the MII spindle before and after treatment with 35 μM H_2_O_2_. Notably, 4-HNE aggregates also co-localised microtubule asters (white arrows). Scale bar = 20 μm. (**b**) γ-tubulin (green) also co-localised with 4-HNE (red) at the poles of the MII spindle before and after treatment with 35 μM H_2_O_2_. (**c–e**) For immunoprecipitation assays, lysates from H_2_O_2_-treated GV and IVM MII oocytes were incubated with protein G Dynabeads conjugated with anti-tubulin antibodies. The Dynabeads were then washed and proteins that bound were eluted for immunoblotting on two mirrored membranes. One membrane (panel 1) was probed with anti-tubulin antibodies confirming the effectiveness of the immunoprecipitation. The alternate membrane was probed with 4-HNE antibodies (panel 2). Whole oocyte lysate was used as a positive control. Antibody-only control (ab only) and preclear elute negative controls were used to confirm specificity of elution. (**f**) PLA between 4-HNE and α-, β- or γ-tubulin revealed fluorescent foci at the MII spindle indicating an intimate association between 4-HNE and tubulins. Scale bar = 20 μm. Nuclei were counterstained with Hoechst (blue). Experiments were performed with three biological replicates with each replicate comprising between 10–30 oocytes pooled from a minimum of three animals. Immunoprecipitation experiments was performed in technical duplicate from between 350–500 oocytes pooled from between 8–12 mice.

Consistent with our previous data, MII oocytes that had received prior exposure to 35 µM H_2_O_2_ at the GV stage of their development presented with strong co-localisation of 4-HNE and α-tubulin within the meiotic spindle and spindle poles. Similar co-localisation, was also present in the untreated control population of MII oocytes (Fig. 6a). 4-HNE labelling also readily co-localised with γ-tubulin in the spindle poles of both H_2_O_2_ (35 µM) treated and untreated MII oocytes (Fig. 6b). However, H_2_O_2_-treated MII oocytes displayed additional foci of co-labelling within microtubule asters (Fig. 6a,b; white arrows). These data implicate the tubulin protein family as primary targets of 4-HNE adduction in the oocyte, a result that was subsequently confirmed *via* immunoprecipitation of α-, β-, and γ-tubulin from H_2_O_2_-treated GV and MII oocyte lysates. The efficacy of this approach in isolating each tubulin isoform was validated by immunoblotting of the captured proteins with isoform specific anti-tubulin antibodies, revealing the anticipated bands at approximately 55 kDa in both GV and MII eluates (Fig. 6c–e). Importantly, no such proteins were detected in any of the negative control samples, including antibody-only, bead-only and precleared lysate. Subsequent probing of each blot with anti-4-HNE antibodies led to strong labelling of equivalent 55 kDa bands in both GV and MII eluates (Fig. 6c–e), thus affirming the susceptibility of all three tubulin isoforms to 4-HNE modification within the female gamete.

**Figure 7:**
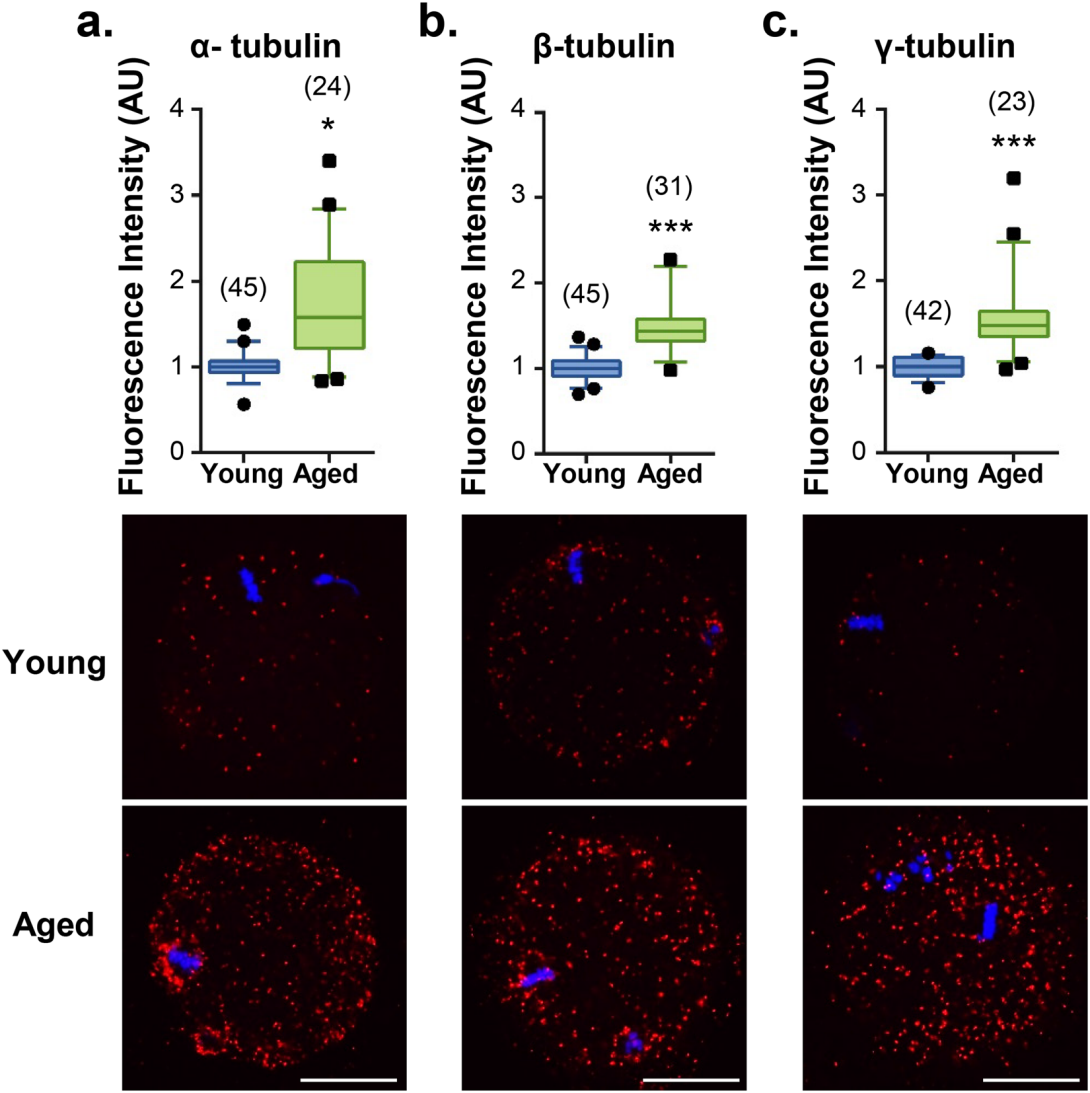
4-HNE adduction to α-, β- and γ-tubulin accumulates in the ageing oocyte. PLA fluorescent foci (red) intensity throughout the entire oocyte was used to compare the extent of α -, β- and γ-tubulin/4-HNE interactions between MII oocytes from young (4 to 6 weeks) and aged (14 months) mice. PLA revealed a significant increase in (**a**) α- (*p* ≤ 0.0420), (**b**) β- (*p* ≤ 0.0002) and (**c**) γ-tubulin (*p* ≤ 0.0003), adduction by 4-HNE in the oocytes of aged mice. Scale bar = 20 μm. Nuclei were counterstained with Hoechst (blue). Box and whisker plots show means as the centreline, box as the 25–75th percentiles and whiskers as the 10–90th percentiles. Ageing assays were performed with n = 3 mice, with each mouse representing an independent technical replicate and with a minimum of 7 oocytes.

As an additional line of evidence to substantiate these data, we utilised proximity ligation assays (PLA), a form of co-localisation in which punctate fluorescent signals are only generated if the two targeted antigens reside within a maximum of 40 nm of each other^72, 73^. Analysis of MII oocytes subjected to PLA with a combination of anti-4-HNE and either anti-α-, β- or γ-tubulin antibodies revealed positive punctate fluorescence distributed throughout the oocyte cytoplasm. Notably, intense PLA fluorescence foci were concentrated in the vicinity of the MII spindle (Fig. 6f). The specificity of PLA labelling was substantiated by the complete absence of any fluorescence in negative controls, including an irrelevant antibody combination (i.e. anti-4-HNE and anti-PIWIL1 antibodies), in addition to the omission of primary antibodies. Importantly, PLA analysis with anti-4-HNE and either anti-α-, β- or γ-tubulin antibodies also revealed a substantial increase in labelling within the cytosol and/or the vicinity of the meiotic spindle in untreated oocytes recovered from aged animals (Fig. 7a–c).

### The nucleophile penicillamine is able to ameliorate the impact of acute treatment with H_2_O_2_ on oocytes

Taken together, our data raise the intriguing prospect that the structural perturbation of tubulin that is induced by 4-HNE adduction may, at least in part, contribute to increased aneuploidy rates observed in oocytes exposed to elevated levels of OS. Consistent with this notion, our molecular modelling of previously reported 4-HNE modified residues of α- and β- tubulin^58, 74, 75^ suggests that the introduction of these adducts does have the potential to elicit significant structural and thus functional changes (Fig. S4). Further, despite sharing only 20% amino acid identity among α-, β-, and γ-tubulin (Fig. S5), it is possible that such changes could similarly impact γ-tubulin.

Such findings prompted us to investigate the potential for chemical amelioration of the negative impact of acute H_2_O_2_ exposure on oocyte quality using anti-oxidant supplementation. Penicillamine was selected as the nucleophile of choice owing to its capacity to covalently bind 4-HNE and thus limit its bio-availability^76^. Furthermore, penicillamine has been shown to reduce the negative effects of 4-HNE on embryo development^29^ and human sperm zona-pellucida binding^59^ by reducing 4-HNE adduction to vulnerable proteins. Thus, the impact of penicillamine supplementation was assessed *via* PBE rates, aneuploidy rates and PLA detection of 4-HNE adducted tubulin in oocytes that have experience OS.

**Figure 8:**
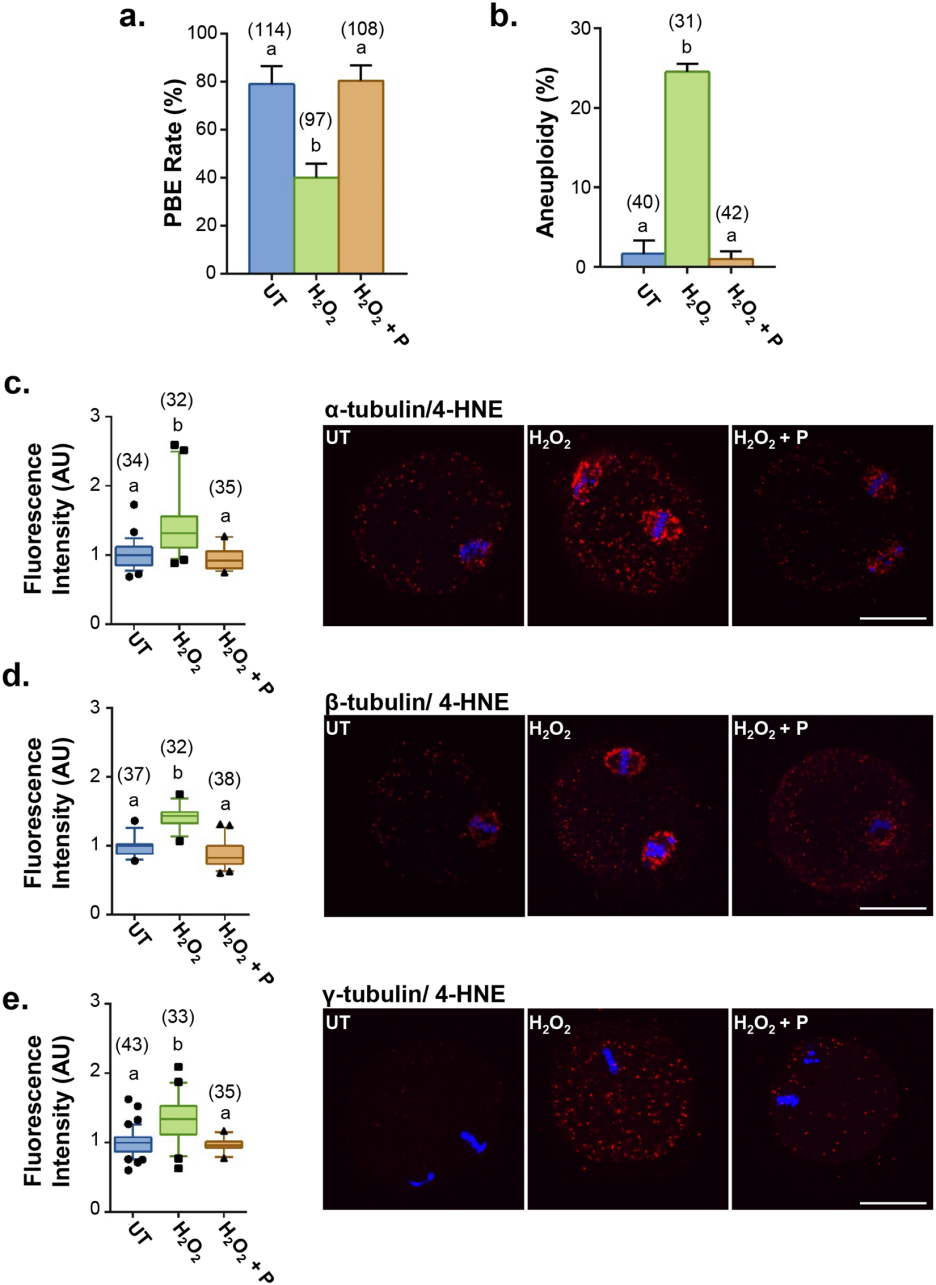
4-HNE adduction can be ameliorated by treatment with penicillamine. GV oocytes were either untreated (UT) or treated for 1 h with either 35 μM H_2_O_2_ (H_2_O_2_) or 35 μM H_2_O_2_ and 1 mM penicillamine (H_2_O_2  _ + P) followed by IVM. Treatment with penicillamine during H_2_O_2_ was able to negate the effect of H_2_O_2_ on (**a**) meiotic completion (*p* ≤ 0.0002) and (**b**) aneuploidy rates (*p* ≤ 0.0001). Error bars represent SEM. (**c**) Upon treatment with H_2_O_2_, fluorescence intensity of PLA (red) showed an increase in 4-HNE adduction to (**c**) α- (*p* ≤ 0.0309) (**d**), β- (*p* ≤ 0.0037) (**e**) and γ-tubulin (*p* ≤ 0.0001) which was ameliorated by treatment with penicillamine during oxidative insult. Scale bar = 20 μm. Nuclei were counterstained with Hoechst (blue). Box and whisker plots shows mean as the centreline, box as the 25–75th percentiles and whiskers as the 10–90th percentiles. PLA was performed with three biological replicates with each replicate comprising between 10–30 oocytes pooled from a minimum of three animals.

Notably, penicillamine treatment was able to significantly reduce the detrimental effect of OS on oocyte. This was best illustrated by assessment of PBE rate, which in the presence of 35 µM H_2_O_2_ reduced the PBE rate to 40%, compared with 79% in untreated controls (*p* ≤ 0.0002; Fig. 8a). The co-incubation of oocytes with penicillamine during H_2_O_2_ exposure completely rescued this pathology with a recovery of PBE rates to 80%, a level that was indistinguishable from that of the control (*p* ≤ 0.0002; Fig. 8a). Aneuploidy rates were similarly ameliorated with penicillamine treatment; from 1.7% aneuploidy rate observed in untreated controls, 24.5% in oocytes exposed to 35 µM H_2_O_2_ alone, and only 1% upon co-treatment with penicillamine (*p* ≤ 0.0001; Fig. 8b).

Further implicating 4-HNE adduction to tubulin as a possible contributor to age-related pathologies was the fact that 35 µM H_2_O_2_ exposure resulted in an increase in 4-HNE adduction to α-, β-, and γ-tubulin as detected *via* PLA fluorescent intensity analysis. Aligning with aneuploidy rates, penicillamine treatment during H_2_O_2_ exposure resulted in the resolution of α- (*p* ≤ 0.0309; Fig. 8c), β- (p ≤ 0.0037; Fig. 8d) and γ-tubulin (*p* ≤ 0.0001; Fig. 8e) adductions to a level that was equivalent to untreated controls.

## Disscussion

An increase in OS within the follicular environment is well known to accompany the process of maternal ageing and contribute to the aetiology of declining oocyte quality in these individuals. While electrophilic lipid aldehydes are well-characterized products of non-enzymatic lipid peroxidation resulting from OS, the role of these aldehydes in the deteriorating quality of maternally aged oocytes has yet to be established. The present study provides evidence that increased OS within the aged ovary, elicits a concomitant increase in the electrophilic aldehyde by-product of 4-HNE, which appears to selectively accumulate within the oocyte. Moreover, our study strongly implicates elevated 4-HNE generation as a key contributor to the ROS mediated deterioration of the ageing oocyte *via* the adduction of key meiotic proteins including members of the tubulin family.

A suite of highly reactive lipid aldehyde species are generated during the progression of lipid peroxidation cascades induced under conditions of OS. Among these, 4-HNE ranks as one of the major cytotoxic aldehydes, and as such, was the target aldehyde for our investigations^63, 64^. At physiological concentrations, 4-HNE is involved in a wide array of biological functions including transcriptional and translational inhibition, enzymatic inactivation as well as the stimulation of signal transduction cascades^69, 77^. In contrast, at the pathological levels induced by OS, the highly electrophilic nature of 4-HNE can result in the formation of stable covalent Michael and Schiff base adducts to nucleophilic functional groups of proteins, such as the side chains of cysteine, lysine and histidine. The formation of these adducts can subsequently result in protein crosslinking, structural perturbation, protein aggregation and inactivation of protein function^52–56^.

Mounting evidence suggests that significant increases in OS occur in the mammalian ovary during maternal ageing^16, 62^. This aligns with a notable elevation in the levels of ROS detected in human follicular fluid from women of advanced maternal age^32, 33^. Similarly, an increase in 4-HNE and several other well defined markers of OS such as nitrotyrosine (NTY), and 8-hydroxy-2′-deoxyguanosine (8-OHdG), have also been identified in the interstitial tissue of the ageing mouse ovary^62^, thus creating a strong precedent for the study of 4-HNE in the deterioration of oocyte quality with age. Our data extends on these findings, revealing an increase in 4-HNE expression within all ovarian cell types upon maternal ageing, including a pronounced accumulation within oocytes and granulosa cells. Aged oocytes are therefore likely exposed to elevated levels of intrinsically generated 4-HNE in addition to exposure from a compromised follicular environment. Consistent with this notion, our data revealed an increase in cytosolic ROS within isolated GV and MII oocytes from female mice of advanced age, which was strongly correlated with elevated 4-HNE production in these cells. The dysregulation of ROS metabolising enzymes with age, such as sirtuin proteins, provides one explanation for the source of ROS detected in our studies^78, 79^. In addition, we were also able to demonstrate that exposure to exogenous H_2_O_2_ at prophase I of oocyte development was able to induce the generation of 4-HNE that propagated throughout meiosis. Taken together, these data lend support to the hypothesis that an age-dependent increase in ROS generation within the ovarian environment results in amplification of the levels of lipid aldehydes, such as 4-HNE, within the oocyte.

Such findings take on added significance in view of the demonstration that exposure to exogenous 4-HNE, or H_2_O_2_, was able to precipitate a similar range of pathologies to those documented in the aged oocyte. A recent study in an equivalent mouse strain (C57Bl/6 × CBA F1) revealed that mis-segregation errors were evenly divided between NDJ and PSSC^65^. Indeed, in our studies treatment with either molecule led to pronounced, dose-dependent increases in spindle and chromosomal deformities, as well as elevated rates of aneuploidy in the form of both NDJ and PSSC. Several studies implicate elevated OS as a major causative agent in these phenotypes^36–39, 80^. For instance, independent research has recapitulated the effect of oocyte-ageing *via* the addition of the oxidizing agent tert-Butyl-hydroperoxide during IVM of mouse oocytes^39^. As we have documented with 4-HNE, this insult was capable of inducing reduced MI and MII spindle size, increased chromosome scattering and clumping on the MII plate as well as an elevation in aneuploidy rates, thus providing compelling evidence for the role of OS in age-related aneuploidy. Furthermore, the induction of OS at prophase I in the oocytes of model species, such as *Drosophila melanogaster*, also precipitates hallmarks of the ageing oocyte such as premature loss of cohesion and consequent chromosome segregation errors^80^. These data demonstrate the vulnerability of oocytes to OS at the developmental stage in which they remain arrested for decades in humans. Similarly, there is also evidence that acute exposure to non-cytotoxic doses of 4-HNE are capable of inducing chromosome segregation errors such as aneuploidy and tetraploidy during mitosis in somatic cells of the normal and malignant colonic epithelial lineage^81, 82^. Such data reinforce the notion that the damage elicited by 4-HNE generation could be a key contributor to the increase in aneuploidy seen with age.

The stability of the dynamic microtubule reorganisation that occurs during meiosis is essential for its faithful completion, with defects in this process, like those observed in our study, leading to elevated rates of aneuploidy^70, 83^. In the present study, H_2_O_2_ treatment precipitated the 4-HNE adduction of three tubulin family members (α, β and γ), thus presenting a possible mechanism by which OS can induce these ageing phenotypes. Indeed, while there are several explanations that could account for the decline in oocyte quality with age (please see below), our ability to document increases in the level of 4-HNE bound tubulin in aged oocytes, supports non-enzymatic, post-translation modification of this protein family as a contributing mechanism. Site-specific 4-HNE adduction to cysteine and lysine residues in the primary structure of α- and β-tubulin have been previously reported to impair tubulin functionality in somatic cells^58, 74, 75^. In fact, such studies revealed that 4-HNE adduction resulted in rapid disappearance of microtubule networks^84^, tubulin crosslinking and inhibition of polymerisation^74, 85^, as well as the spontaneous generation of tubulin dimers^81^. In accounting for these findings, our own molecular modelling of the known sites for 4-HNE adduction within tubulin indicate that this form of modification is likely to cause both changes in the tertiary structure of the protein and importantly, alter the surface characteristics of the tubulin monomers. These structural perturbations are likely to affect the critical protein-protein interactions required for microtubule formation and therefore, 4-HNE adduction is likely to disrupt tubulin polymerisation. Interestingly, despite the conservation of only 20% amino acid sequence identity between α-, β-, and γ-tubulin, we were able to show that each of these isoforms are vulnerable to 4-HNE adduction upon exposure of oocytes to either H_2_O_2_ or 4-HNE. Since γ-tubulin holds an essential role in microtubule nucleation, the novel finding that it is also susceptible to 4-HNE modification offers a plausible explanation for the spindle deformities, microtubule disorganisation and ensuing aneuploidy observed in this, and other studies of aged oocytes^3–5, 86, 87^. Undoubtedly, determining the fate of 4-HNE adducted tubulin and its rate of turnover within the mouse oocyte presents exciting avenues for future research.

While our study supports the role of 4-HNE in the age-dependent deterioration of oocyte quality *via* adduction of tubulins, it should also be noted that a number of additional proteins within the oocyte, also proved vulnerable to 4-HNE adduction. Although the identity of these additional proteins was not investigated directly, it is perhaps noteworthy that they are of similar molecular weight to proteins that have previously been reported as being sensitive to 4-HNE adduction and are also important for the successful completion of meiosis. These proteins include, SDHA, protein kinase A, tubulin polymerization promoting protein family member 2, protein kinase A and actin^29, 58, 75, 88^.

As an additional line of enquiry, our study also presents preliminary data demonstrating the capacity of the highly nucleophilic and reactive thiol, penicillamine, to prevent OS elicited damage to oocytes *via* averting tubulin- 4-HNE adduction, meiotic arrest and aneuploidy. Importantly, the ability of penicillamine to combat the impact of acute H_2_O_2_ exposure affords support for the notion that this damage is mediated by electrophilic aldehyde(s). Penicillamine has been demonstrated to significantly reduce free 4-HNE expression because the 4-HNE Michael addition kinetics for the penicillamine substrate, is far greater than that for any amino acid side chain, thus quenching the aldehyde *via* covalent interaction and limiting its bio-availability^29, 76^. Moreover, penicillamine can also act to chelate transition metals, including the Fenton metals, iron and copper, which are responsible for catalysing the lipid peroxidation cascades that promote the production of these aldehydes^76^. Administration of other antioxidants such as resveratrol, N-acetyl-L-cysteine (NAC), vitamin C and E have also proven successful in preserving oocyte quality from ageing mice. In this context, several studies have reported almost complete conservation of mouse oocyte quality upon administration of antioxidants from weaning, or for several months prior to oocyte isolation^89–93^. Moreover, it has also been reported that antioxidant therapy can assist with the recovery of oocyte quality following oxidative insult over shorter periods of time. Indeed, administration of melatonin during human ovarian stimulation in preparation for IVF reduced OS parameters and improved blastocyst quality and development^94^. Additionally, media supplementation with the antioxidants cystine and cysteamine during IVM resulted in decreased aneuploidy and improved embryo development after maternal restraint stress-induced OS^95^ and maternal ageing^96^. In the context of this study, our data suggest that antioxidant supplementation may be able to prevent the generation of aldehydes such as 4-HNE and/or restrict their capacity to damage key meiotic proteins, including those of the tubulin family.

Notably, an increase in the generation of lipid aldehydes is unlikely to be the sole detrimental impact of age-related oxidative stress on oocyte quality. For instance, H_2_O_2_ is able to directly induce DNA double stand breaks (DSB) within the oocyte genome^97^ and these lesions have been associated with chromosome misalignment and spindle defects at MII^98^. Accordingly, an increase in the DNA DSB burden has also be detected in maternally aged oocytes^99–101^. Furthermore, H_2_O_2_ is also known to cause pervasive depolarisation of mitochondria, which can also lead to increases in spindle disruption^37^. Indeed, as we have documented previously, maternal ageing is well known to be accompanied by mitochondrial dysfunction^22, 23^. As a final consideration in defining the causative factors that contribute to increased OS in maternally aged oocytes, it is notable that the oxidative defence capacity of surrounding granulosa and cumulus cells have also been reported to decline with age^102–104^. While the removal of cumulus cells in our study enabled us to focus on the selective response of the oocyte to OS, we acknowledge the merit of exploring the protective capacity afforded by granulosa and cumulus cells against 4-HNE in future studies.

Taken together, our data have established a causative link between OS induced aldehyde generation and the decrease in oocyte quality seen in the aged oocyte. We have presented evidence for accelerated 4-HNE production in aged ovarian tissue as well as in aged oocytes, and have implicated increased 4-HNE expression in the deterioration of oocyte quality with the induction of increased defects in spindle assembly, chromosome alignment and elevated aneuploidy. We have also identified a potential mechanistic link between elevated OS, 4-HNE modification of α-, β-, and γ-tubulins, and increased aneuploidy rates within the oocyte as is commonly seen with increasing maternal age. In doing so, this research may help inform our understanding of the molecular basis of age related decline in female fertility and the development of rationale therapeutic intervention strategies to combat this pressing issue.

## Materials and Methods

### Materials

All chemicals and reagents used were of research grade and were supplied by ThermoFisher Scientific (Waltham, MA, USA) or Sigma-Aldrich (St. Louis, MO, USA) unless otherwise specified. Please see Table S2 for details on primary antibodies used for immunolocalisation and immunoblotting and immunoprecipitation assays. Appropriate HRP-conjugated secondary antibodies were obtained from Santa Cruz Biotechnology and Sigma-Aldrich. Alexa Fluor 488-conjugated goat anti-mouse (Cat #A-11001), 633-conjugated goat anti-rabbit (Cat #A-21070) and 555-conjugated goat anti-human (Cat #A-21433) antibodies were purchased from ThermoFisher Scientific.

### Ethics statement

Research animals in this study were handled, monitored and euthanised in accordance with NSW Animal Research Act 1998, NSW Animal Research Regulation 2010 and the Australian Code for the Care and Use of Animals for Scientific Purposes 8^th^ Ed. as approved by the University of Newcastle Animal Care and Ethics Committee (approval number A-2011-162). C57/BL6xCBA F1 hybrid female mice were bred and held at the institutes’ Central Animal House with food and water *ad libitum*. Animals were housed under a 12 h light/12 h dark cycle at a constant temperature of 21–22 °C and euthanised immediately before use *via* cervical dislocation.

### Oocyte collection

Oocytes were isolated as previously described^105^. Briefly, mice between 4 to 6 weeks of age were administered intraperitoneal injections of 7.5 IU equine chorionic gonadotropin (eCG) (Intervet, Sydney NSW, Australia) to stimulate *in vivo* oocyte maturation to mature GV stage. Forty-eight hours following eCG injection, animals were euthanised and ovaries were removed. Pre-ovulatory follicles were repeatedly punctured with a 27-gauge needle to release mature GV oocytes as cumulus-oocyte-complexes into pre-warmed (37 °C) M2 media supplemented with 2.5 µM milrinone to maintain GV arrest. Media droplets were kept under mineral oil at all times, to prevent evaporation (Mineral oil; embryo tested). Only oocytes with an intact layer of cumulus cells were recovered. Cumulus cells were mechanically removed *via* repeated aspiration with a narrow pipette at 37 °C.

### Induction of OS

To examine the consequence of OS on oocyte quality, GV oocytes were treated with either H_2_O_2_ (0–100 µM) for 1 h or 4-HNE (0–50 µM) for 2 h at 37 °C in M2 media supplemented with 2.5 µM milrinone and under mineral oil. Following treatment, GV oocytes were either processed immediately or underwent IVM.

### *In vitro* maturation

For IVM, oocytes were washed out of milrinone by aspiration through four 50 μl droplets of MEM α media (Cat #11900024, ThermoFisher Scientific) supplemented with 20% (v/v) foetal calf serum, 50 U/ml penicillin, and 50 μg/ml streptomycin before being placed into a single-well IVF dish (Cat. #353653), containing 500 μl of MEM α media. Oocytes were cultured at 37 °C in an atmosphere of 5% CO_2_ for 16 h to generate a pool of MII stage oocytes. All media and mineral oil were equilibrated in appropriate conditions for a minimum of three hours before use. Following IVM, oocyte maturation was scored, with GV oocytes being identified by the presence of a nuclear envelope and nucleolus, MI oocytes identified by the absence of the nuclear envelope and nucleolus, MII oocytes identified *via* the presence of the first polar body, and degenerative oocytes identified *via* cytoplasmic fragmentation^106, 107^.

### Penicillamine treatment

In an effort to ameliorate the effect of OS on oocyte quality, the nucleophile D-penicillamine was utilised to limit bioavailability of lipid aldehydes^29, 59, 60^. For this purpose, GV oocytes were exposed to 35 μM H_2_O_2_ for 1 h at 37 °C in the presence of 1 mM D-penicillamine (Cat #P4875-1G, Sigma-Aldrich), prior to IVM in MEM α at 37 °C in 5% CO_2_. Following IVM, oocytes were scored for PBE rates and then processed for assessment of aneuploidy and 4-HNE adduction to tubulin *via* PLA as described below.

### Measurement of cytosolic ROS generation

Where required, chloromethyl-2′,7′-dichlorodihydro-fluorescein diacetate (CM-H2DCFDA; Cat #C6827, ThermoFisher Scientific) was used as an indicator of cytosolic ROS generation. For this purpose, oocytes were incubated in for 1 h at 37 °C in CM-H2DCFDA (4 μM) prepared in either M2 media supplemented with milrinone (GV oocytes) or in MEM α media under an atmosphere of 5% CO_2_ (MII oocytes). Bright field and epifluorescence images were acquired using a Nikon Biostation IM (Nikon Instruments Inc., Melville, NY, USA).

### Immunocytochemistry

Following treatment and/or IVM, oocytes were washed in phosphate buffered saline (PBS) containing 3 mg/ml polyvinylpyrrolidone (PBS/PVP) before being fixed in 2% paraformaldehyde (w/v) diluted in PBS/0.5% Triton-X (v/v) for 30 min and prepared for immunostaining. Fixed oocytes were blocked in 7% goat serum (v/v) and 1% BSA (w/v) prepared in PBS/0.1% Tween-20 (PBST) for 1 h at room temperature. The cells were then incubated with either anti-4-HNE, α-tubulin, γ-tubulin, or CREST antibodies diluted to appropriate concentrations in 1% BSA (w/v)/PBST overnight at 4 °C. After washing in 1% BSA (w/v)/PBST, oocytes were incubated with appropriate AlexaFluor conjugated secondary antibodies (diluted 1:1000 in 1% BSA (w/v)/PBST) for 1 h at room temperature. All experiments included secondary only controls in which the primary antibody was substituted with 1% BSA (w/v)/PBST. An additional control was also utilised to confirm the specificity of antibody labelling whereby anti-4-HNE antibodies were pre-absorbed with excess 4-HNE prior to use. For this purpose, 4-HNE (150 µM) was conjugated to L-Lysine (900 µM) in PBST for 6 h at room temperature. The L-Lysine-4-HNE conjugate was then co-incubated with anti-4-HNE antibody overnight at 4 °C. The primary antibody was then substituted with the L-Lysine-4-HNE-anti-4-HNE antibody suspension (Fig. S2b). Oocytes were counterstained with Hoechst 33258 (20 µg/ml) diluted in PBS/PVP for 15 min at room temperature. Finally, oocytes were mounted on Menzel Gläser microscope slides (Thermo Fisher Scientific) in Citifluor Glycerol Solution AF2 (Cat #AGR1321, Citifluor Ltd., London, UK). To ensure accurate fluorescence quantification, all oocytes used in a single experimental replicate were collected, treated, fixed and permeabilised concurrently. Immunostaining protocols were also performed with all treatment groups concurrently with equivalent antibody concentrations, volumes and incubation times. Oocytes were then mounted in equal volumes of Citifluor to minimise quenching of fluorescence signals during image capture.

### Immunofluorescent labelling of ovarian tissue

Ovarian tissue section was prepared as described previously^108^ prior to being de-paraffinised by a series of three × 5 min xylene washes, and rehydrated with successive ethanol washes; 2 × 100%, 90%, 70%, 50%, and H_2_O. Antigen retrieval was performed in 10 mM Tris (pH 9) by microwaving for 10 min (1100 W) and slides were then blocked with 7% goat serum (v/v) in 1% BSA (w/v)/PBST for 1 h at room temperature. Primary antibody incubations were conducted overnight at 4 °C in the presence of anti-4-HNE antibody diluted to an appropriate concentration in 1% (w/v) BSA/PBST. Following repeated washes in PBST, slides were incubated with goat anti-rabbit AlexaFluor 594 conjugated secondary antibody for 1 h at room temperature. After washing with PBST, slides were counterstained with the nuclei marker 4′-6-diamindino-2-phenylindole (DAPI, 0.5 μg/ml in PBS) for 2 min. Finally, sections were rinsed in PBST and mounted in Citifluor and images were acquired using an Olympus FV1000 confocal microscope.

### Assessment of oocyte aneuploidy status

The aneuploidy status of oocytes was assessed following treatment with either H_2_O_2_ or 4-HNE as previously described^109, 110^. Briefly, following H_2_O_2_ or 4-HNE exposure at the GV stage of development, oocytes were subjected to IVM. The resultant populations of oocytes were then incubated with 200 μM monastrol in MEM α for 2 h at 37 °C in 5% CO_2_. Oocytes were fixated and kinetochores were immunostained with anti-CREST antibodies as described above.

### Proximity ligation assays

Proximity ligation assays (PLA) were performed on fixed oocytes using the Duolink *In Situ* Red Starter Kit Mouse/Rabbit as per the manufacturer’s instructions (Cat #DUO92101-1KT, Sigma-Aldrich). Briefly, oocytes were blocked with Duolink blocking solution for 30 min at 37 °C, incubated with appropriate pairs of primary antibodies (i.e. anti-4-HNE and either anti-α-tubulin, anti-β-tubulin and anti-γ-tubulin antibodies) diluted in Duolink antibody buffer overnight at 4 °C. Labelled oocytes were then washed thoroughly with PBS/PVP prior to incubation with oligonucleotide-conjugated secondary antibodies (PLA probes) for 1 h at 37 °C. After additional washes, the ligation and amplification of PLA probes was conducted in accordance with the manufacturer’s instructions. Finally, oocytes were counterstained with Hoechst 33258 and images were acquired using an Olympus FV1000 confocal microscope as described above. Negative control incubations used to confirm specificity of the assay included antibody pairs targeting proteins that would not be expected to interact (i.e. anti-PIWIL1 and anti-α-tubulin antibodies) as well as the omission of each primary antibody.

### SDS-PAGE and immunoblotting

Electrophoretic resolution of oocyte proteins was conducted by SDS-PAGE using standard procedures with minor modifications^111^. Briefly, protein was extracted from isolated oocytes *via* direct incubation in SDS extraction buffer comprising 2% SDS (w/v), 10% sucrose (w/v) in 0.1875 M Tris, pH 6.8 and supplemented with ProteCEASE protease inhibitors (Cat #786‐326, G-Biosciences, MO, USA) and boiling (100 °C for 5 min). Entire protein lysates recovered from 100 oocytes were diluted into SDS-PAGE loading buffer containing 2% β-mercaptoethanol and bromophenol blue before being resolved on NuPage 4–12% Bis-Tris gels (Cat #NP0321BOX, ThermoFisher Scientific) and transferred using an XCell Blot Module (Cat #EI9051, ThermoFisher Scientific) onto nitrocellulose membranes (Cat #10600002, GE Healthcare, Buckinghamshire, UK). Membranes were blocked by incubation in 3% BSA (w/v)/Tris-buffered saline (TBS; pH 7.4) and 0.1% Tween-20 (TBST) for 2 h at room temperature before being incubated with anti-4-HNE, anti-α-tubulin, anti-β-tubulin, anti-γ-tubulin or anti-GAPDH antibodies each diluted in 1% BSA (w/v)/TBST overnight at 4 °C. Membranes were washed three times with TBST and incubated with horseradish peroxidase-conjugated secondary antibody diluted into 1% BSA (w/v)/TBST for 1 h. Following three washes in TBST, labelled proteins were detected using an enhanced chemiluminescence kit (Cat #RPN2106, GE Healthcare) and visualised using ImageQuant LAS 4000 (Fujifilm, Tempe, AZ, USA). Densitometry analysis was performed with ImageJ software (National Institutes of Health, MD, USA) using the ECL signal intensity generated over the entire lane. Data was normalised against a GAPDH protein loading control.

### Immunoprecipitation

Immunoprecipitation was conducted as previously described^112^ in order to validate proteins targeted for 4-HNE adduction. For each immunoprecipitation assay, 12.5 µg (equivalent to 500 oocytes) were lysed in 200 μl of 3-[(3-cholamidopropyl)dimethylammonio]-1-propanesulfonate (CHAPS) lysis buffer comprising 137 mM NaCl, 10% glycerine (v/v), 10 mM CHAPS, 10 mM HEPES and supplemented with ProteCEASE protease inhibitors. Lysis was conducted for 2 h at 4 °C with constant rotation and solubilised protein separated *via* centrifuged (4 °C, 20,000 × g, 20 min). The oocyte proteins so isolated were subjected to a pre-clearing step whereby they were incubated with unlabelled protein G Dynabeads for 1 h at 4 °C with constant rotation. Simultaneously, 10 μg of antibody was incubated with protein G magnetic Dynabeads (Cat #10004D, ThermoFisher Scientific) for 2 h at 4 °C. The bead suspension was then washed prior to cross-linking of bound antibodies *via* incubation with 5 mM bis(sulfosuccinimidyl) suberate (BS_3_) (Thermo Fisher Scientific) in 20 mM HEPES at room temperature on rotation for 30 min. An excess of 12.5 μl of 1 M Tris (pH 7.5) was applied and the incubation continued for a further 15 min to quench the cross-linking reaction. The antibody-bead complexes were then incubated with pre-cleared oocyte protein at 4 °C overnight with constant rotation. The bead complexes were subsequently washed with PBS and bound proteins were eluted by heating at 70 °C for 5 min in a solution of 30 μl of SDS-PAGE loading buffer, prior to being resolved by SDS-PAGE and prepared for immunoblotting as described above.

### Mass spectrometry

To identify proteins with potential 4-HNE modifications, mass spectrometry was performed as previously described^113^. Briefly, target proteins were excised from SDS-PAGE gels and peptides generated through tryptic digestion. Tryptic peptides were fractionated *via* reverse phase nano-LC (Dionex Ultimate 3000 RSLCnano, Idstein, Germany) and sequenced by tandem mass spectrometry (LC–MS/MS) on an electrospray ionisation 3D Ion Trap Mass Spectrometer (AmaZon ETD, Bruker Daltonik, Bremen, Germany). Peptide sequences were aligned against SwissProt mouse databases (*Mus musculus*) using an in-house licensed MASCOT server (version 2.3.02, Matrix Science, London, UK). Peptide thresholds were set requiring a false-positive rate 0.05% and a MASCOT score greater than 40. Those meeting the criteria were manually validated to ensure accurate y- and b-ion detection with overlapping sequence coverage.

### Confocal Imaging

All cell images were captured using high resolution confocal microscopy on an Olympus FV1000 confocal microscope. Oocytes were imaged under a 60× oil immersion lens with a z-resolution of 0.5 μm (CREST) or 2 μm (tubulin, 4-HNE). Fluorochromes were imaged sequentially to avoid bleed-through. Oocytes from each treatment group were imaged with identical parameters on the same day to minimise fluorescence fading.

### Measurement of Fluorescent Intensities

For GV oocytes, images chosen for quantification were those captured through the mid-section of the oocyte; positioned to incorporate the centre of the nucleolus and thus encompass the centre of the nucleus as well as the cytoplasm. Similarly, for MII oocytes, the images used for quantification were captured through the mid-section of the oocyte; positioned to incorporate the centre of the MII plate and encompass the spindle. The entire area within the oocyte was used for ICC quantification. Fluorescence intensity for immunocytochemistry was measured using ImageJ (National Institutes of Health, MD, USA). The integrated fluorescence intensity of a mid-section (encompassing the DNA) of the whole oocyte, was determined and the background fluorescence was measured at four locations on the image, and averaged. For determination of fluorescence intensity in captured images, the corrected total cell fluorescence (CTCF), or normalised fluorescence, was used as described in the following equation; CTCF = Integrated fluorescence intensity - (area of selected cell × average background fluorescence). This measurement considers differences in the size of oocytes via correction of the background staining intensity for the size of the cell. Data collected from individual experimental replicates were normalised to appropriate untreated controls.

### Statistical analysis

Statistical analysis was performed using two-tailed unpaired Student’s *t*-tests and one-way analysis of variances (ANOVA) with Tukey’s post-hoc multiple comparison using Graphpad Prism 7 software (San Diego, CA, USA). A *p* value of <0.05 was considered significant. Experiments were performed in biological triplicate unless otherwise stated. Statistical analyses were performed using the mean of each biological replicate. Data are expressed as means ± S.E.M or as box and whisker plots with means as the centreline, boxes as the 25–75th percentiles and whiskers as the 10–90th percentiles.

## Electronic supplementary material

Supplementary Information
