# Supplementary material for: The lipid peroxidation product 4-hydroxynonenal contributes to oxidative stress-mediated deterioration of the ageing oocyte
Source: Sci Rep. 2017 Jul 24;7:6247. doi: 10.1038/s41598-017-06372-z (PMC5524799; doi:10.1038/s41598-017-06372-z)
Supplement: Supplementary file 1 — Supplementary Information [file 41598_2017_6372_MOESM1_ESM.pdf]

The lipid peroxidation product 4-hydroxynonenal contributes to oxidative stress-mediated deterioration of the ageing oocyte

*Bettina P. Mihalas[1], Geoffry N. De Iuliis[1], Kate. A. Redgrove[1], Eileen A. McLaughlin[1,2], *Brett Nixon[1]

1. Priority Research Centre for Reproductive Science, School of Environmental and Life Sciences, University of Newcastle, Callaghan, New South Wales, Australia

2. School of Biological Sciences, University of Auckland, Auckland, New Zealand

***Corresponding Author(s):**

Ms. Bettina Mihalas

Professor Brett Nixon

Priority Research Centre for Reproductive Science

School of Environmental and Life Sciences

The University of Newcastle, University Drive, Callaghan, NSW, 2308

T: +612 4921 6977

F: +612 4921 6308

E: Bettina.Mihalas@uon.edu, Brett.Nixon@newcastle.edu.au

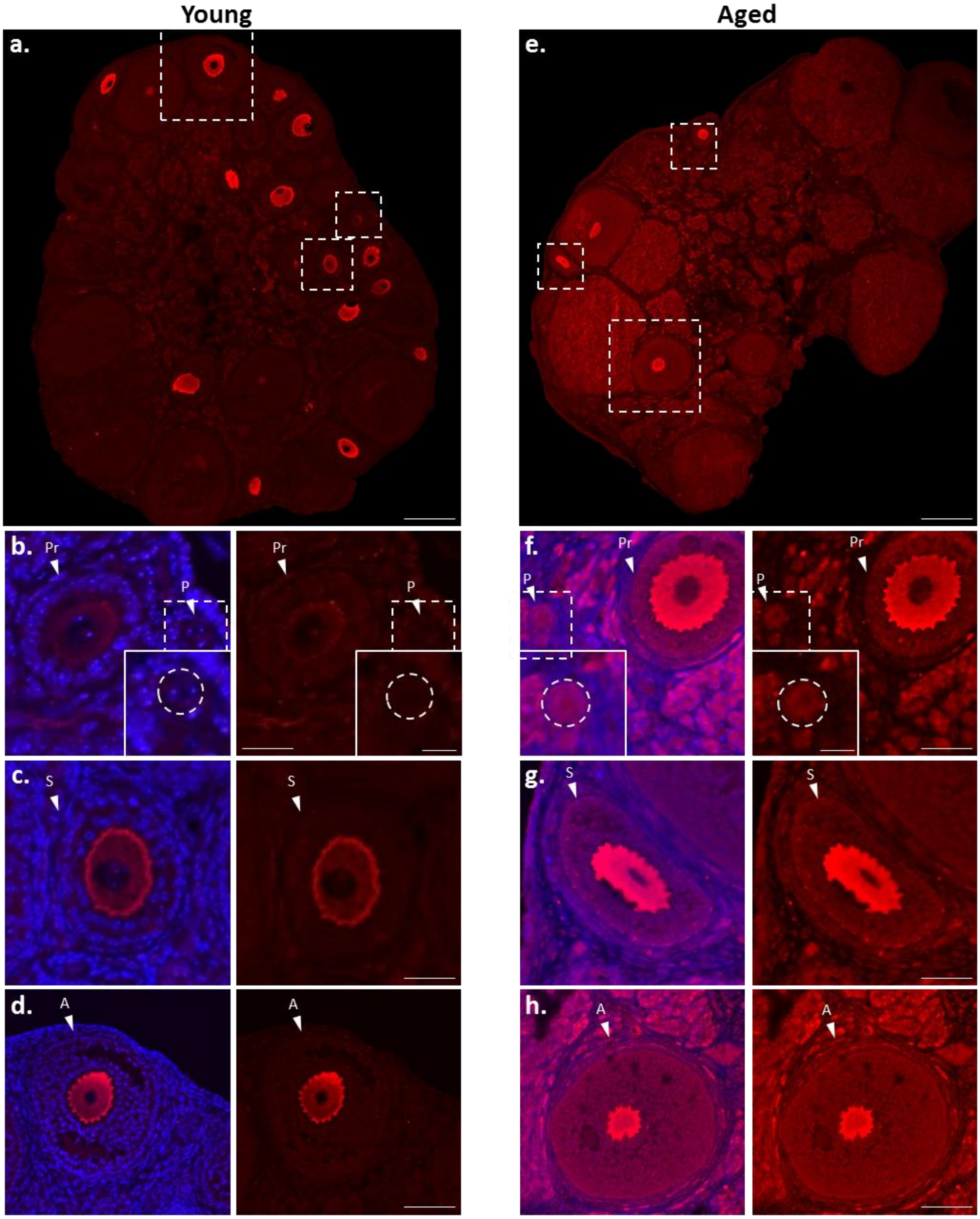

Fig. S1: **4-HNE is a by-product of lipid peroxidation in the mouse ovary.** The production and localisation of 4-HNE between young (4 to 6 weeks) and aged (14 months) mouse ovaries was determined using immunofluorescence**. (a)** 4-HNE expression (red) was observed at basal levels within all cell types within the young ovary (scale bar = 200 µm) including oocytes in **(b)** primordial (P), primary (Pr, scale bar = 20 µm), **(c)** secondary (S, scale bar = 40 µm) and **(d)** antral follicles (A, scale bar = 80 µm). **(e)** 4-HNE generation was also observed in all cell types within the aged ovary (scale bar = 300 µm) including oocytes in **(f)** primordial, primary (scale bar = 20 µm) **(g)** secondary (scale bar = 40 µm) and **(h)** antral follicles (scale bar = 80 µm). Nuclei were counterstained with DAPI (blue). Immunofluorescence assays were performed in both technical and biological triplicate.

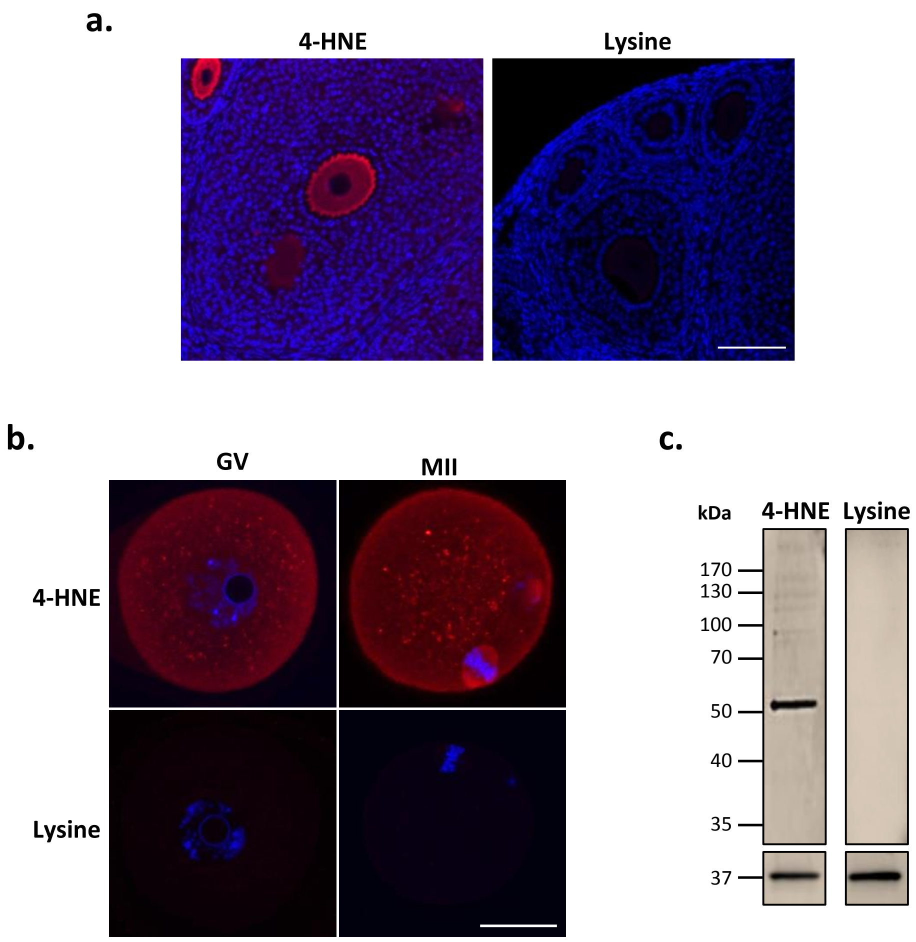

Fig. S2: **4-HNE antibody specificity.** L-Lysine-4-HNE bound anti-4-HNE antibody controls (lysine) revealed no unspecific primary or secondary antibody binding during **(a)** Immunofluorescence (scale bar = 40 µm) or **(b)** immunocytochemistry (scale bar = 20 µm) compared to unbound anti-4-HNE antibody (red). Nuclei were counterstained with DAPI (Immunofluorescence) or Hoechst (immunocytochemistry) (blue). Antibody specificity was also confirmed for immunoblotting, which also revealed no unspecific primary or secondary antibody binding in L-Lysine-4-HNE bound anti-4-HNE antibody controls (lysine). Immunoblots were stripped and re-probed with GAPDH as a loading control.

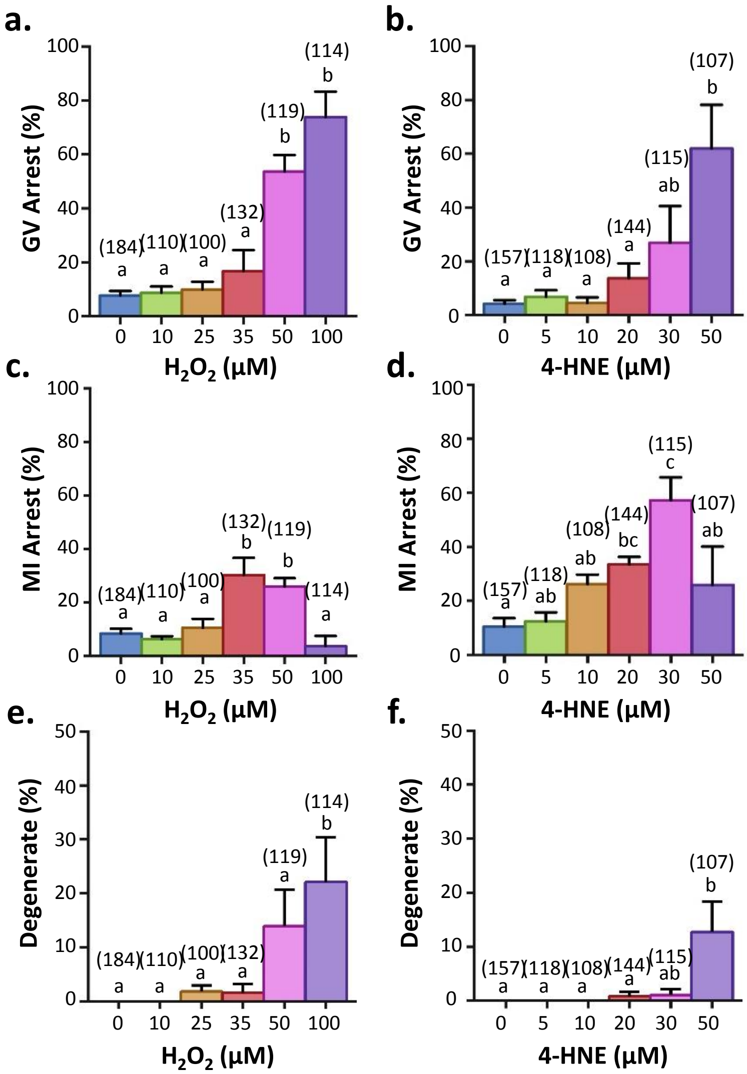

Fig. S3: **Acute exposure to H_2O_2 and 4-HNE at GV stage causes meiotic arrest and degeneration.** Oocytes at GV stage were treated with either H_2O_2 for 1 h or 4-HNE for 2 h prior to IVM for 16 h. For the purpose of this analysis, GV oocytes were identified by the presence of a nuclear envelope and nucleolus, MI oocytes by the absence of the nuclear envelope and nucleolus and degenerative oocytes were identified *via* cytoplasmic fragmentation. **(a)** A dose-depended increase in GV arrest was observed after H_2O_2 (one-way ANOVA; $p \leq 0.0011$) and **(b)** 4-HNE (one-way ANOVA; $p \leq 0.0037$) treatment. **(c)** A dose-dependent increase following a significant decrease was also observed in MI arrest after H_2O_2 (one-way ANOVA; $p \leq 0.0456$) and **(d)** 4-HNE (one-way ANOVA; $p \leq 0.0420$) treatment. **(e)** Furthermore, a dose-depended increase in oocyte degeneration was observed after H_2O_2 (one-way ANOVA; $p \leq 0.0037$) and **(f)** 4-HNE (one-way ANOVA; $p \leq 0.0003$) treatment. Error bars represent SEM. IVM experiments were performed with a minimum of five replicates with each replicate containing between 20-50 oocytes pooled from a minimum of three animals.

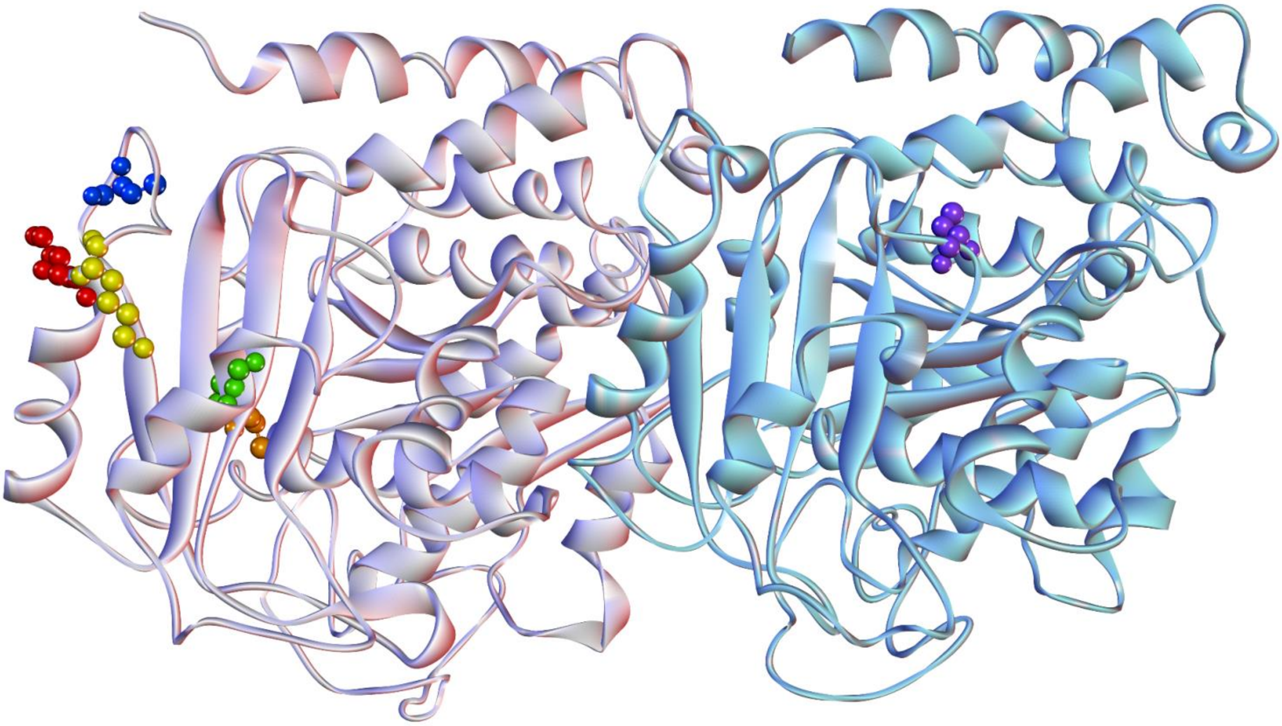

Fig. S4: **α- and β-tubulin dimer homology model with confirmed 4-HNE modifications.** α- tubulin (white) 4-HNE modifications have been identified on Cys295 (green), Lys336 (red), Lys338 (yellow), Cys347 (blue) and Cys376 (orange) as well as on β-tubulin (blue) on Cys303 (purple)[46,62,63].

```
Q71U36 TBA1A_HUMAN      1   -MRECISIHVGQAGVQIGNACWELYCLEHGIQPDGQMPSDKTIGGGDDSFNTFFSETGAG   59
Q9H4B7 TBB1_HUMAN       1   -MREIVHIQIGQCGNQIGAKFWEMIGEEHGIDLAGSDRGASAL--QLERISVYYNEAYGR   57
P23258 TBG1_HUMAN       1   MPREIITLQLGQCGNQIGFEFWKQLCAEHGISPEGIVEEFATE--GTDRKDVFFYQADDE   58
                              ** : :::**.* ***   *:    ****.  *      :     :  ..:: ::

Q71U36 TBA1A_HUMAN     60   KHVPRAVFVDLEPTVIDEVRTGTYRQLFHPEQLITG--KEDAANNYARGHYTIGKEIIDL   117
Q9H4B7 TBB1_HUMAN      58   KYVPRAVLVDLEPGTMDSIRSSKLGALFQPDSFVHG--NSGAGNNWAKGHYTEGAELIEN   115
P23258 TBG1_HUMAN      59   HYIPRAVLLDLEPRVIHSILNSPYAKLYNPENIYLSEHGGGAGNNWASG-FSQGEKIHED   117
                            :::****::**** .:..: ..    *::*:.:  .    .*.**:* * :: * :: :

Q71U36 TBA1A_HUMAN    118   VLDRIRKLADQCTGLQGFLVFHSFGGGTGSGFTSLLMERLSVDYGKKSKLEFSIYPAP-Q   176
Q9H4B7 TBB1_HUMAN     116   VLEVVRHESESCDCLQGFQIVHSLGGGTGSGMGTLLMNKIREEYPDRIMNSFSVMPSP-K   174
P23258 TBG1_HUMAN     118   IFDIIDREADGSDSLEGFVLCHSIAGGTGSGLGSYLLERLNDRYPKKLVQTYSVFPNQDE   177
                            ::: : : :: .  *:** : **:.******: : *::::   * .:    :*: *  :

Q71U36 TBA1A_HUMAN    177   VSTAVVEPYNSILTTHTTLEHSDCAFMVDNEAIYDICRRNLDIERPTYTNLNRLIGQIVS   236
Q9H4B7 TBB1_HUMAN     175   VSDTVVEPYNAVLSIHQLIENADACFCIDNEALYDICFRTLKLTTPTYGDLNHLVSLTMS   234
P23258 TBG1_HUMAN     178   MSDVVVQPYNSLLTLKRLTQNADCVVVLDNTALNRIATDRLHIQNPSFSQINQLVSTIMS   237
                            :* .**:***::*: :  :::*. . :** *:   *.   *.:  *:: ::*:*:.  :*

Q71U36 TBA1A_HUMAN    237   SITASLRFDGALNVDLTEFQTNLVPYPRIHFPLATYAPVISAEKA-YHEQLSVAEITNAC   295
Q9H4B7 TBB1_HUMAN     235   GITTSLRFPGQLNADLRKLAVNMVPFPRLHFFMPGFAPLTAQGSQ-QYRALSVAELTQQM   293
P23258 TBG1_HUMAN     238   ASTTTLRYPGYMNNDLIGLIASLIPTPRLHFLMTGYTPLTTDQSVASVRKTTVLDVMRRL   297
                            . *::**: * :* **  : ..::* **:** :  ::*: :  .    .  :* :: .

Q71U36 TBA1A_HUMAN    296   FEPANQMVKCDPR---HGKYMACCLLYRGDVVPKDVNAAIATIKTKRTIQFVDWCPTGFK   352
Q9H4B7 TBB1_HUMAN     294   FDARNTMAACDLR---RGRYLTVACIFRGKMSTKEVDQQLLSVQTRNSSCFVEWIPNNVK   350
P23258 TBG1_HUMAN     298   LQPKNVMVSTGRDRQTNHCYIAILNIIQGEVDPTQVHKSLQRIRERKLANFIPWGPASIQ   357
                            ::  * *.  .     .  *::   : :*.:  .:*.  :  : ::.   *: * * ..:

Q71U36 TBA1A_HUMAN    353   VGINYQPPTVVPGGDLAKVQRAVCMLSNTTAIAEAWARLDHKFDLMYAKRAFVHWYVGEG   412
Q9H4B7 TBB1_HUMAN     351   VAVCDIPPRGL--------SMAATFIGNNTAIQEIFNRVSEHFSAMFKRKAFVHWYTSEG   402
P23258 TBG1_HUMAN     358   VALSRKSPYLPSAH-----RVSGLMMANHTSISSLFERTCRQYDKLRKREAFLEQFRKED   412
                            *.:    *             :  ::.* *:* . : *  .::. :  :.**:. :  *.

Q71U36 TBA1A_HUMAN    413   MEEGEFSEARED---MAALEKDYEEVGVDSVEGEGEEE-GEEY---------   451
Q9H4B7 TBB1_HUMAN     403   MDINEFGEAENN---IHDLVSEYQQFQDAKAVLEEDEEVTEEAEMEPEDKGH   451
P23258 TBG1_HUMAN     413   MFKDNFDEMDTSREIVQQLIDEYHAATRPDYISWGTQEQ-------------   451
                            *  .:*.*   .   :  * .:*.     .      :*
```

Fig. S5: **Amino acid sequence alignment between α-, β- and γ-tubulin represent a 20% sequence homology.** An asterisk (*) represents residues conserved between three tubulin isoforms, a colon (:) represents conserved substitution of residues between tubulin isoforms and a full stop (.) represents semi-conserved substitutions such as amino acids with similar characteristics. α-tubulin 4-HNE modifications have been identified on Cys295 (green), Lys336 (red), Lys338 (yellow), Cys347 (blue) and Cys376 (orange) as well as on β- tubulin on Cys303 (purple)[46,62,63].

***Table S1*: LC–MS/MS protein identification (ID) of the predominant 55 kDa protein(s) targeted for adduction by 4-HNE.**

Protein ID (gene symbol)	Mascot score	MW [kDa]	No. Peptides
Actin (ACTB)	152.1	41.7	3
Tubulin alpha (TBA3)	81.1	49.9	2
Tubulin beta (TBB4A)	78.1	49.9	2

***Table S2:* Antibodies used for immunocytochemistry (ICC), immunofluorescence (IF), proximity ligation assays (PLA), immunoprecipitation (IP) and immunoblotting (IB).**

Antibody	Species	Concertation	Dilution/ concentration	Source and Cat#
4-HNE	Rabbit polyclonal	1 mg/ ml	1:50 (IF, ICC, PLA)<br>1:500 (IB)	Cat # HNE11-S, Jomar Diagnostics
4-HNE	Mouse monoclonal	0.1 mg/ ml	1:50 (IB)	Cat # ab48506, Abcam
α-tubulin	Mouse monoclonal	1 mg/ ml	1:400 (ICC, PLA)	Cat # A11126 ThermoFisher
α-tubulin	Rabbit monoclonal	1 mg/ ml	10 μg (IP)	Cat # ab52866, Abcam
α-tubulin	Mouse monoclonal	6 mg/ ml	1:2000 (IB)	Cat # T5168, Sigma-Aldrich
β-tubulin	Rabbit polyclonal	1 mg/ ml	10 μg (IP)	Cat # ab6046, Abcam
β-tubulin	Mouse monoclonal	0.2 mg/ ml	1:100 (ICC, PLA)<br>1:500 (IB)	Cat# sczsc-5274, Santa Cruz
γ-tubulin	Rabbit polyclonal	0.6 mg/ ml	10 μg (IP)	Cat # ab84355, Abcam
γ-tubulin	Mouse monoclonal	5 mg/ ml	1:1000 (ICC, PLA)<br>1:5000 (IB)	Cat # T6557, Sigma-Aldrich
CREST	Human monoclonal	1 mg/ ml	1:400 (ICC)	Cat # 90C-CS1058, Fitzgerald
GAPDH	Rabbit polyclonal	5 mg/ ml	1:5000 (IB)	Cat # G9545, Sigma-Aldrich
